# Histone deacetylase 10 structure and molecular function as a polyamine deacetylase

**DOI:** 10.1038/ncomms15368

**Published:** 2017-05-18

**Authors:** Yang Hai, Stephen A. Shinsky, Nicholas J. Porter, David W. Christianson

**Affiliations:** 1 Roy and Diana Vagelos Laboratories, Department of Chemistry, University of Pennsylvania, 231 South 34th Street, Philadelphia, Pennsylvania 19104-6323, USA

## Abstract

Cationic polyamines such as spermidine and spermine are critical in all forms of life, as they regulate the function of biological macromolecules. Intracellular polyamine metabolism is regulated by reversible acetylation and dysregulated polyamine metabolism is associated with neoplastic diseases such as colon cancer, prostate cancer and neuroblastoma. Here we report that histone deacetylase 10 (HDAC10) is a robust polyamine deacetylase, using recombinant enzymes from *Homo sapiens* (human) and *Danio rerio* (zebrafish). The 2.85 Å-resolution crystal structure of zebrafish HDAC10 complexed with a transition-state analogue inhibitor reveals that a glutamate gatekeeper and a sterically constricted active site confer specificity for *N*^8^-acetylspermidine hydrolysis and disfavour acetyllysine hydrolysis. Both HDAC10 and spermidine are known to promote cellular survival through autophagy. Accordingly, this work sets a foundation for studying the chemical biology of autophagy through the structure-based design of inhibitors that may also serve as new leads for cancer chemotherapy.

## Abstract (web summary)

Polyamines bind to nucleic acids and their function is regulated by reversible acetylation. Here, the authors show that histone deacetylase 10 is a polyamine deacetylase and present its crystal structure with a bound polyamine transition state analogue inhibitor.

Polyamines such as putrescine, spermidine and spermine are ubiquitous and essential in all living systems^1^,^2^,^3^. Typically present at millimolar concentrations, these cellular polycations bind to nucleic acids and other anionic macromolecules to stabilize structure and regulate function in myriad biological processes. Polyamine metabolism is highly regulated and consists of an elaborately orchestrated balance of cellular uptake, intracellular transport, biosynthesis and catabolism (Fig. 1a)^4^. Dysregulation of polyamine metabolism is frequently associated with cancer and other hyperproliferative diseases, and enzymes of polyamine metabolism are emerging drug targets^5^,^6^.

A critical strategy for the regulation of polyamine function is reversible acetylation. This strategy is analogous to the regulation of protein function by reversible lysine acetylation through reactions catalysed by histone acetyltransferases and histone deacetylases (HDACs, also known more generally as lysine acetyltransferases and lysine deacetylases). In eukaryotes, this branch of polyamine metabolism is partially compartmentalized in the nucleus (Fig. 1a). Spermidine is acetylated at the *N*^8^ position by an *N*-acetyltransferase in the cell nucleus^7^ and then exported to the cytosol, where it is deacetylated by a metal-dependent *N*^8^-acetylspermidine deacetylase, also known as polyamine deacetylase (PDAC)^8^,^9^. The substrate specificity of PDAC is very strict, as it does not deacetylate cytosolic *N*^1^-acetylspermidine or *N*^1^-acetylspermine^10^. Selective inhibition of PDAC activity in HeLa cells increases *N*^8^-acetylspermidine levels but not acetylated histone levels, so PDAC activity is distinct from HDAC activity^11^.

To date, the identity of the cytosolic PDAC in eukaryotes has remained elusive. However, selective inhibitors have been developed that block mammalian PDAC activity^11^,^12^,^13^. Our recent studies of a bacterial PDAC^14^ belonging to the arginase-deacetylase superfamily^15^,^16^ led us to suggest that the enigmatic PDAC might be one of the two cytosolic class IIb Zn^2+^-dependent HDACs, HDAC6 or HDAC10.

We recently reported the X-ray crystal structures of both catalytic domains (CDs) of HDAC6, showing that a conserved active site lysine serves as a gatekeeper in CD1, in that it dictates specificity for carboxy-terminal acetyllysine substrates bearing a negatively charged α-carboxylate group^17^. In all other human HDACs, this residue is otherwise conserved as leucine or methionine, except for HDAC10 in which it is a glutamate (Fig. 1b and Supplementary Fig. 1). Accordingly, we hypothesized that this strictly conserved glutamate could similarly serve as a gatekeeper to confer specificity for positively charged acetylpolyamine substrates. HDAC10 is highly expressed in the liver, spleen and kidney^18^,^19^, enriched in the cytoplasm^19^ and resistant to inhibition by sodium butyrate^20^. These properties match the tissue distribution, subcellular localization and inhibitor sensitivity of mammalian PDAC^8^,^9^,^10^,^11^,^12^,^13^. Thus, we further hypothesized that HDAC10 might be a PDAC. Here we report enzymological studies conclusively demonstrating that HDAC10 is a robust PDAC and a poor lysine deacetylase. In addition, we report the 2.85-Å resolution crystal structure of HDAC10 complexed with a polyamine transition state analogue inhibitor. This structure reveals that the conserved glutamate gatekeeper and a sterically constricted active site confer specificity for *N*^8^-acetylspermidine hydrolysis and disfavour acetyllysine hydrolysis.

## Results

### HDAC10 is a PDAC

To determine steady-state kinetic parameters for the hydrolysis of a variety of acetylpolyamine and acetyllysine substrates, we prepared full-length recombinant HDAC10 from human (*H. sapiens*, hHDAC10) and zebrafish (*D. rerio*, zHDAC10). The CDs of these orthologues are highly similar based on 63%/79% sequence identity/similarity; only full-length constructs yielded soluble proteins. We first assayed the lysine deacetylase activity of these constructs against two commercially available aminomethylcoumarin (AMC)-conjugated fluorogenic acetyllysine peptides, GAK(ac)-AMC and RGK(ac)-AMC (molecular structures of all substrates are shown in Supplementary Fig. 2). Both hHDAC10 and zHDAC10 exhibited very low activity with these peptides (Fig. 2 and Supplementary Table 1). These measurements were consistent with earlier studies reporting that HDAC10 activity was not measurable using fluorogenic peptide substrates^21^. We also tested activity with 11 non-fluorogenic acetyllysine substrates using a liquid chromatography–mass spectrometry (LC–MS)-based discontinuous assay^17^ to avoid potential steric clashes that might occur with the bulky AMC group. These substrates included K(ac)NL and GAK(ac)NLQ, which mimic the peptide segment containing K73 in the proposed HDAC10 substrate MutS homologue 2 (MSH2)^22^. Again, we observed no or very low catalytic activity (Fig. 2 and Supplementary Table 1). In comparison with its closest relative, the cytosolic isozyme HDAC6 (ref. 17), HDAC10 was clearly not an efficient lysine deacetylase.

**Figure 1:**
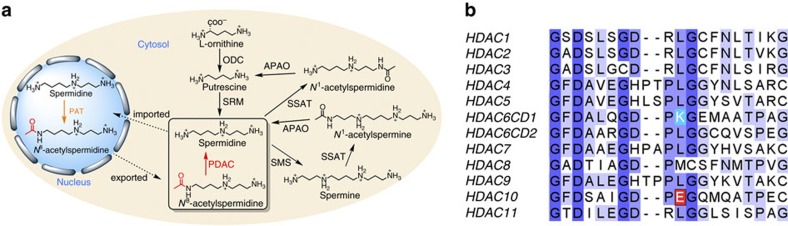
Eukaryotic polyamine metabolism and HDAC gatekeepers. (**a**) APAO, *N*^1^-acetylpolyamine oxidase; ODC, ornithine decarboxylase; PAT, *N*^8^-spermidine acetyltransferase; PDAC, *N*^8^-acetylspermidine deacetylase; SMS, spermine synthetase; SRM, spermidine synthase; SSAT, spermidine/spermine acetyltransferase. (**b**) Sequence alignment of the 11 metal-dependent human HDACs (the two CDs of HDAC6, CD1 and CD2, are listed separately). Gatekeeper K353 of hHDAC6 CD1 is highlighted in sky blue and gatekeeper E272 of hHDAC10 is highlighted in red. In all other isozymes, this residue is leucine or methionine and exerts no electrostatic influence on substrate binding.

**Figure 2:**
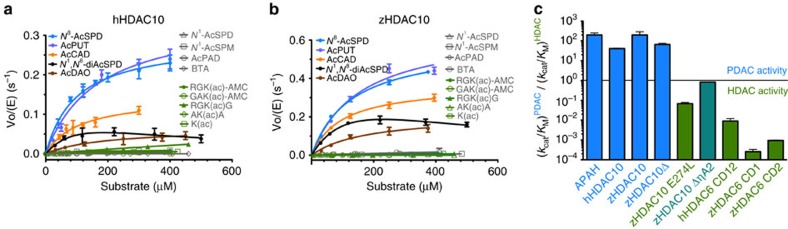
Catalytic activity of hHDAC10 and zHDAC10. (**a**,**b**) Steady-state kinetics of hHDAC10 and zHDAC10 assayed with acetylpolyamines and acetyllysine-containing peptides. There is a clear preference for acetylpolyamine hydrolysis. AcCAD, acetylcadaverine; AcDAO, *N*-(aminooctyl)acetamide; AcPUT, acetylputrescine; AcPAD, *N*-(3-aminopropyl)acetamide; BTA, *N-*butylacetamide; *N*^1^-AcSPD, *N*^1^-acetylspermidine; *N*^1^-AcSPM, *N*^1^-acetylspermine; *N*^8^-AcSPD, *N*^8^-acetylspermidine; *N*^1^,*N*^8^-diAcSPD, *N*^1^,*N*^8^-diacetylspermidine. (**c**) Ratio of catalytic efficiencies (*k*_cat_/*K*_M_) for PDAC activity measured with *N*^8^-acetylspermidine and HDAC activity measured with RGK(ac)-AMC. APAH, hHDAC10 and zHDAC10 exhibit a clear catalytic preference for PDAC activity. The E274L mutation converts zHDAC10 from a PDAC into an HDAC and the zHDAC10 ΔηA2 mutant is a bifunctional PDAC-HDAC. APAH, acetylpolyamine amidohydrolase; hHDAC6 CD12, human HDAC6 construct containing both CDs; hHDAC10, human HDAC10; zHDAC10, zebrafish HDAC10; zHDAC10Δ, proteolytically nicked zHDAC10 used for the crystal structure determination; zHDAC6 CD1 or CD2, zebrafish HDAC6 CD1 or CD2. Data represent mean±s.d. (*n*=3).

We next tested the PDAC activity of hHDAC10 and zHDAC10 against several polyamine substrates using the LC–MS assay (Fig. 2, Supplementary Fig. 3 and Supplementary Table 1). Both enzymes exhibited optimal catalytic activity and specificity for the hydrolysis of *N*^8^-acetylspermidine. For hHDAC10 and zHDAC10, *k*_cat_/*K*_M_=2,900±200 and 4,600±300 M^−1^ s^−1^, respectively; in comparison, *N*^1^-acetylspermidine was a poor substrate with *k*_cat_/*K*_M_=24±5 and 7±2 M^−1^ s^−1^, respectively. Substrate specificity trends observed for zHDAC10 and hHDAC10 matched those of the previously reported mammalian *N*^8^-acetylspermidine deacetylase activity^9^,^10^,^11^. As a positive control, we measured the *N*^8^-acetylspermidine deacetylase activity of *Mycoplana ramosa* acetylpolyamine amidohydrolase (APAH), which exhibits a similar preference for PDAC activity over acetyllysine deacetylase activity but which also exhibits broader polyamine substrate specificity^23^. As a negative control, we examined the *N*^8^-acetylspermidine deacetylase activity of human and zebrafish HDAC6 (ref. 17). HDAC6 preferentially catalyses the deacetylation of the peptide substrate RGK(ac)-AMC with a catalytic efficiency >100-fold greater than that for the deacetylation of *N*^8^-acetylspermidine. Therefore, HDAC6 is clearly an acetyllysine deacetylase and HDAC10 is an *N*^8^-acetylspermidine deacetylase. It is notable that the molecular function of HDAC10 coincides with the cellular function of the cytosolic PDAC indicated in Fig. 1a.

Although the PDAC activity of zHDAC10 and hHDAC10 was highest with *N*^8^-acetylspermidine, substantial catalytic activity was also observed with acetylcadaverine and acetylputrescine, as well as *N*-(8-aminooctyl)acetamide; in contrast, the shorter substrates *N*-butylacetamide and *N*-(3-aminopropyl)acetamide exhibited minimal or no activity, respectively (Fig. 2). These results suggested that the ideal length of a PDAC substrate ranged 8–12 atoms long. The N4 amino group of *N*^8^-acetylspermidine was required for optimal activity in comparison with *N*-(8-aminooctyl)acetamide, which lacks the secondary amino group. Even so, the amino group could be located at N4 or N5, based on the substantial catalytic activity measured with acetylcadaverine and acetylputrescine. However, the amino group could not be at N3, based on the lack of substantial catalytic activity with *N*^1^-acetylspermidine and *N*-(3-aminopropyl)acetamide. In addition, given the attenuated catalytic activity measured for *N*^1^,*N*^8^-diacetylspermidine, we concluded that the positive charge of the *N*^1^-amino group of *N*^8^-acetylspermidine contributed to, but was not critical for, enzyme–substrate recognition. In comparison with HDAC10, the bacterial PDAC APAH exhibited broad substrate specificity with all polyamine substrates except for *N*-butylacetamide; it also exhibited weak lysine deacetylase activity (Supplementary Table 1 and Supplementary Fig. 3).

To test our hypothesis that E274 is a gatekeeper conferring substrate specificity for *N*^8^-acetylspermidine, we prepared E274L zHDAC10. In accord with our hypothesis, this substitution diminished *N*^8^-acetylspermidine deacetylase activity by 20-fold and enhanced acetyllysine deacetylase activity by ∼100-fold (Supplementary Fig. 3 and Supplementary Table 1). Thus, the single-point mutation E274L completely reversed the substrate specificity of zHDAC10, converting it from a PDAC into an HDAC (Fig. 2c).

### Crystal structure of HDAC10

To provide a molecular foundation for understanding the PDAC activity of HDAC10, we determined the 2.85 Å-resolution crystal structure of Y307F zHDAC10 complexed with the trifluoromethylketone inhibitor 7-[(3-aminopropyl)amino]-1,1,1-trifluoroheptan-2-one (AAT, Supplementary Fig. 2), which mimics *N*^8^-acetylspermidine^24^. This inhibitor binds as a transition state analogue with *K*_i_=0.7±0.2 μM (Supplementary Fig. 4). We treated full-length zHDAC10 with trypsin to yield a heterodimeric complex, designated zHDAC10Δ, comprising the amino-terminal PDAC domain and the C-terminal pseudo-deacetylase (ΨDAC) domain. The proteolytically nicked zHDAC10Δ construct retains substantial catalytic activity (Supplementary Table 1). Both proteolysis and the introduction of the deactivating Y307F mutation facilitated crystallization.

**Figure 3:**
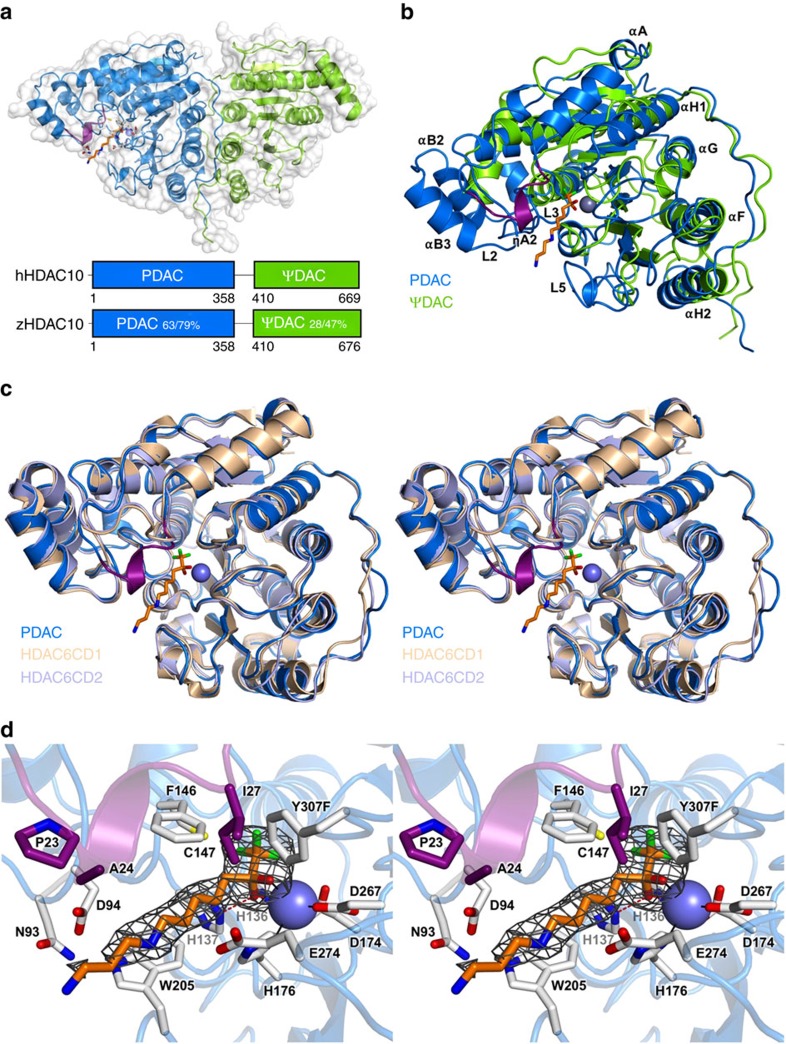
Crystal structure of the Y307F zHDAC10Δ–AAT complex. (**a**) The catalytic PDAC domain and the smaller, noncatalytic ΨDAC domain assemble with butterfly-like architecture. The unique 3_10_-helix ηA2 (P^23^ACE) found in the L1 loop of HDAC10 orthologues is purple. Percentages indicate sequence identity/similarity between zebrafish and human HDAC10 domains. The inhibitor AAT (stick figure, C=yellow, N=blue) binds to the PDAC domain. (**b**) Superposition of the PDAC domain (blue) with the ΨDAC domain (green). Selected secondary structure elements are labelled: helix ηA2 is purple; helices αF, αG and the loop connecting helices αH1 and αH2 mediate domain–domain interactions; helices αB2 and αB3, as well as loops L1–L5, comprise and flank the active site of PDAC but are absent in ΨDAC (for clarity, only loops L2, L3 and L5 are labelled). Zn^2+^ is a blue sphere. (**c**) Stereo view image showing the superposition of zHDAC10 PDAC domain (blue), zHDAC6 CD1 (wheat, PDB 5EEF) and zHDAC6 CD2 (light blue, PDB 5EFH). The 3_10_-helix ηA2 inserted in loop L1 (purple) is unique to zHDAC10 and serves to constrict the PDAC active site. (**d**) Stereo view image showing the simulated annealing omit map of AAT bound in the PDAC active site contoured at 3*σ*. Selected active site residues are indicated; hydrogen bonds are indicated by dashed red lines and metal coordination interactions are represented by solid black lines. Zn^2+^ is a blue sphere.

The crystal structure of the Y307F zHDAC10Δ–AAT complex (Fig. 3a) revealed an overall butterfly-like architecture with a pseudo-two-fold symmetry axis perpendicular to several α-helices defining the PDAC-ΨDAC domain interface. Each domain adopted the α/β-fold first observed in the crystal structure of arginase^25^ and subsequently observed in the HDAC family^26^. However, the ΨDAC domain lacked helices ηA1–A2, αA, αB2–αB3 and active site loops L1–L5 (Fig. 3b). Interdomain interactions were mediated by α-helices αF and αG, loop LH (which connects αH1 and αH2) and the C-terminal tail of each domain, burying ∼1,700 Å^2^ surface area. The 48-residue linker was missing in the electron density, presumably due to proteolysis. HDAC10 PDAC-ΨDAC heterodomain assembly resembles HDAC6 heterodomain assembly and HDAC homodomain assembly in *Schizosaccharomyces pombe* Clr3 (Supplementary Fig. 5)^27^,^28^.

The tertiary structure of the PDAC domain was similar to both CD1 (PDB 5EEF) and CD2 (PDB 5EFH) of HDAC6 (0.78 Å root mean squared deviation (r.m.s.d.) for 355 Cα atoms; 0.88 Å r.m.s.d. for 352 Cα atoms, zCD2) (Fig. 3c). A close-up view of the active site tunnel revealed a canonical HDAC active site at the base of the tunnel, with AAT binding in an extended conformation (Fig. 3d). The trifluoroketone moiety of AAT formed a gem-diolate, making asymmetric coordination interactions with Zn^2+^ (O1-Zn^2+^ and O2-Zn^2+^ coordination distances of 2.1 and 2.5 Å, respectively); hydroxyl group O2 also hydrogen bonded with H136 and H137. The binding of AAT thus mimicked the tetrahedral intermediate and its flanking transition states for the hydrolysis of *N*^8^-acetylspermidine. This binding mode was similar to that observed in the APAH–AAT and zHDAC6 CD2–AAT complexes^17^,^29^.

The structure of zHDAC10 allowed us to study substrate specificity determinants: in particular, the active site was much more constricted compared with other isozymes such as HDAC6. On one side of the active site, gatekeeper E274 was close (3.7 Å) to the N4 atom of AAT (Fig. 3d). Although this interatomic separation was too long for a hydrogen bond interaction, it was sufficiently close for a favourable electrostatic interaction. The conversion of HDAC10 from a PDAC into an HDAC through the E274L mutation (Fig. 2) demonstrated that this electrostatic interaction is important for PDAC specificity.

**Figure 4:**
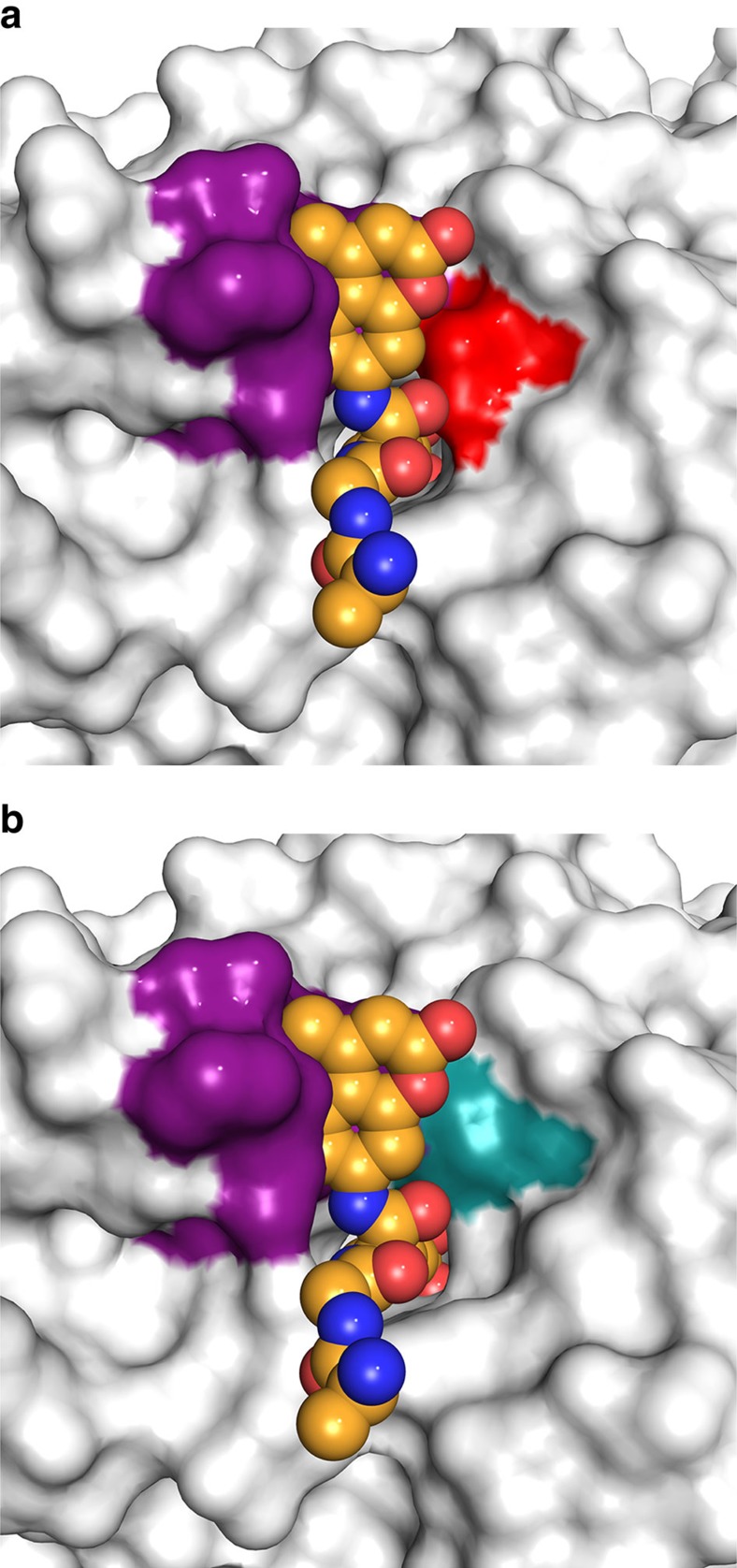
Constriction of the zHDAC10 active site by gatekeeper E274 and the ηA2 helix. (**a**) Crystal structure of zHDAC10 with a bound substrate based on superposition of the structure of the H574A zHDAC6 CD2–RGK(ac)-AMC complex (PDB 5EFN). Atoms of zHDAC6 CD2 are omitted for clarity; note that the arginine side chain of substrate RGK(ac)-AMC is disordered and not shown. The docked substrate suggests that unfavourable steric clashes will occur between the backbone carbonyl of acetyllysine and E274 (red) and between the AMC group (which mimics the residue at the +1 position adjacent to the scissile acetyllysine residue) and the ηA2 helix (purple). (**b**) Same structure as **a**, except that the E274L mutation is introduced (teal) as described in Methods. The E274L mutation widens the active site, alleviating the steric clash with the backbone carbonyl of the acetyllysine substrate, which in turn introduces HDAC activity.

Opposite E274, there was an additional constriction contributing to PDAC specificity: a unique one-turn 3_10_-helix ηA2 (P^23^ACE) resulting from a two-residue insertion plus a two-residue mutation in the L1 loop relative to zHDAC6, effectively lengthening the narrow active site tunnel by ∼5 Å (Fig. 3c,d). This four-residue segment is universally conserved in HDAC10 orthologues with only minor variations, appearing predominantly as PECE (Supplementary Fig. 1). The steric protrusion formed by this segment favours the binding of long acetylpolyamine substrates (Fig. 3c) and disfavours the binding of shorter acetyllysine substrates (Fig. 4). Removal of the 3_10_-helix ηA2 insertion yielded construct zHDAC10 ΔηA2, which exhibited 15-fold decreased PDAC activity and 16-fold increased HDAC activity (Supplementary Table 1, Fig. 2 and Supplementary Fig. 3). Therefore, both gatekeeper E274 and the 3_10_-helix ηA2 insertion promote PDAC activity and suppress HDAC activity.

**Figure 5:**
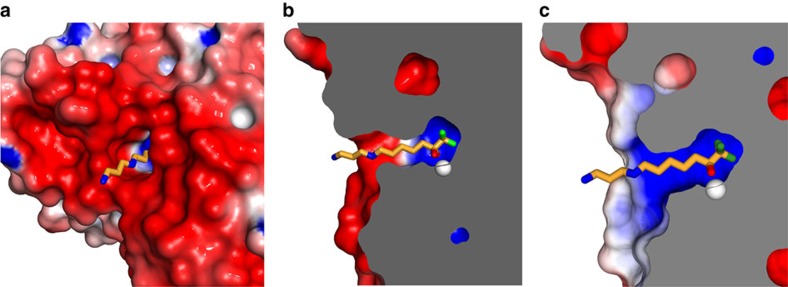
Electrostatic surface potential of HDAC10. (**a**) A negatively charged protein surface surrounding the active site tunnel is a conserved feature of HDAC10 PDAC orthologues based on the conservation of aspartate and glutamate residues (Supplementary Fig. 1). Colour coding is as follows: -5kT–5kT, red–blue. (**b**) Cut-away view of the zHDAC10 active site showing the transition between the negative electrostatic surface potential of the outer active site and the positive electrostatic potential of the inner active site. (**c**) In comparison, no particular electrostatic profile characterizes the protein surface surrounding the active site of the related cytosolic lysine deacetylase, HDAC6 CD2, complexed with AAT (PDB 5EFH).

Although the structural basis of HDAC10 selectivity for *N*^8^-acetylspermidine over acetyllysine-containing peptide substrates appeared straightforward, the structural basis of selectivity for *N*^8^-acetylspermidine over *N*^1^-acetylspermidine was more subtle to discern. The active site tunnel in the PDAC domain was surrounded by a vast surface of negative electrostatic potential (Fig. 5a,b and Supplementary Fig. 6), resulting in large part from conserved aspartate and glutamate residues (Supplementary Fig. 1). This electrostatic surface feature is unique to HDAC10 orthologues and contrasts with that of the lysine deacetylase HDAC6 CD2 (Fig. 5c). However, the surface of the HDAC10 PDAC domain became less anionic and ultimately cationic, at the base of the active site tunnel (Fig. 5b). The binding of *N*^1^-acetylspermidine would place the positively charged N4 amino group closer to this cationic surface, which would disfavour *N*^1^-acetylspermidine binding relative to *N*^8^-acetylspermidine binding. The strict substrate regioselectivity of HDAC10 for *N*^8^-acetylspermidine contrasts with the broad substrate specificity of bacterial APAH, which has a glutamate residue (E117) to stabilize the positively charged N4 amino group through a salt bridge (Supplementary Fig. 7).

The crystal structure of the Y307F zHDAC10Δ–AAT complex revealed that the primary amino group at the end of AAT is disordered (Fig. 3d), but it would be located near the side chains of N93, D94 and A24. Interestingly, D94 corresponds to acetyllysine binding residues D101 of hHDAC8 and S568 of hHDAC6 CD2 (refs 17, 30, 31). To study the influence of N93 and D94 on substrate binding and catalysis in zHDAC10, we prepared the N93A and D94A mutants. Neither mutant exhibited substantial differences in steady-state kinetic parameters (Supplementary Table 1 and Supplementary Fig. 3). Thus, neither N93 nor D94 are required for enzyme–substrate recognition and catalysis.

The ΨDAC domain of HDAC10 has no known catalytic function, although it may play a role in directing cytoplasmic enrichment^19^. Neither the Zn^2+^ ligands nor catalytic residues were conserved in the ΨDAC domain (Fig. 3b). The overall fold of the ΨDAC domain was most similar to that of the PDAC domain, as well as the CDs of HDAC6 (∼2.1 Å r.m.s.d. for ∼213 Cα atoms in each). In contrast with the PDAC domain, the ΨDAC domain exhibited low amino acid sequence identity and greater divergence across species (for example, 28% sequence identity between zebrafish and human proteins) (Fig. 3a and Supplementary Fig. 1); however, α-helices αG and αH at the interdomain interface were highly conserved (Supplementary Fig. 5).

## Discussion

Our enzymological and X-ray crystallographic results suggest that E274 of zHDAC10 is conserved in HDAC10 orthologues as a gatekeeper to establish specificity for a cationic substrate, just as gatekeeper K330 is conserved in the corresponding position of zHDAC6 CD1 to establish specificity for an anionic substrate^17^. This gatekeeper is necessary but not sufficient for full activity and selectivity: both zHDAC10 and zHDAC6 CD1 have additional steric bulk opposite the gatekeeper that further constricts the active site. In zHDAC10, the 3_10_-helix ηA2 provides this steric bulk; in hHDAC6 CD1 (ref. 17), F105 and Y225 provide this steric bulk. Regardless, the gatekeeper appears to be a ‘hot spot' for dictating substrate specificity.

With regard to the substrate specificity of HDAC10, a sizeable array of steady-state kinetic measurements clearly demonstrated that HDAC10 is a PDAC (Fig. 2 and Supplementary Table 1). Even so, HDAC10 is proposed to deacetylate the cytosolic proteins Hsc70/Hsp70 and MSH2 (refs 22, 32). The deacetylation of Hsc70/Hsp70 by HDAC10 is not characterized at the molecular level, so we cannot comment on this possibility. Although the deacetylation of K73 of MSH2 correlates with HDAC10 levels, enzyme catalysis has not been directly demonstrated^22^; moreover, our steady-state kinetic measurements showed that HDAC10 does not catalyse the deacetylation of MSH2-based peptide substrates K(ac)NL and GAK(ac)NLQ (Supplementary Table 1). Intriguingly, however, we demonstrated that the MSH2-based substrate GAK(ac)NLQ is efficiently deacetylated by HDAC6 (Supplementary Table 1 and Supplementary Fig. 3), which is also known to interact with and deacetylate MSH2 *in vivo*^33^. Our *in vitro* results are more consistent with HDAC6 being the MSH2 deacetylase. As a point of speculation, perhaps the very low levels of lysine deacetylase activity measured for some but not all peptide substrates (Supplementary Table 1) are responsible for the effect of HDAC10 on MSH2 acetylation status.

**Figure 6:**
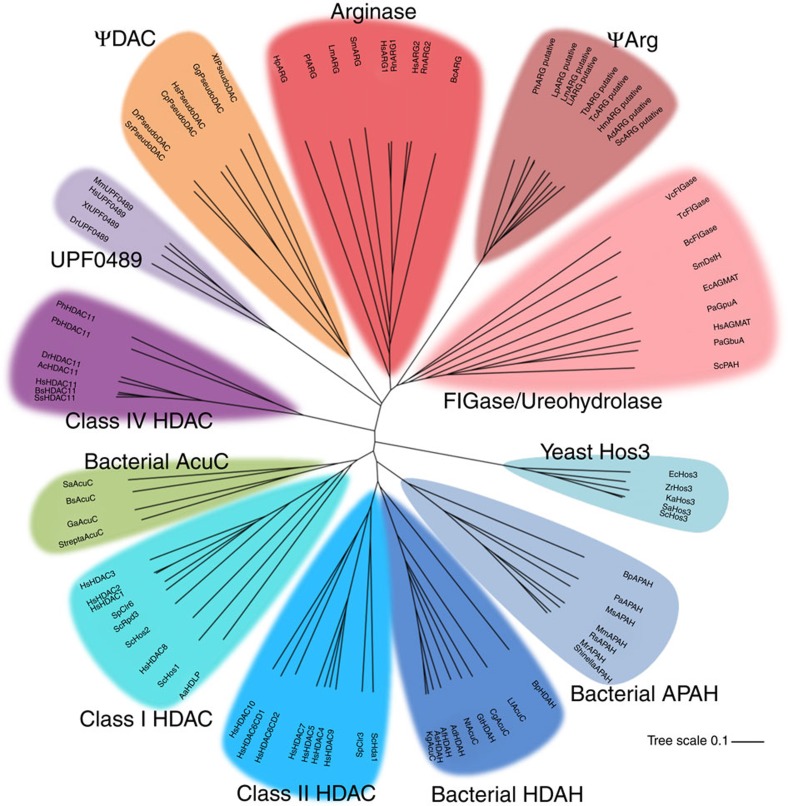
Unrooted phylogenetic tree of the arginase-deacetylase family. The scale of the branches indicates percent amino acid difference. Twelve clades are evident: arginases, pseudo-arginases (ΨArg), formiminoglutamases (FIGase) and ureohydrolases, yeast Hos3 homologues, bacterial APAHs, bacterial histone deacetylase-like amidohydrolases (HDAH), class II HDACs, class I HDACs, bacterial acetoin utilization proteins (AcuC), class IV HDACs, uncharacterized protein family UPF0489 and ΨDAC. Acronyms are defined and UniProt or NCBI accession numbers are listed in Supplementary Table 5.

As previously mentioned, HDACs adopt the same α/β-fold first observed in the crystal structure of arginase, a binuclear manganese metalloenzyme that catalyses the hydrolysis of arginine to form ornithine plus urea^15^,^25^. This evolutionary relationship was unexpected, as there is very low amino acid sequence identity between HDACs and arginases. However, identical α/β-folds (β-strand order 21387456) and the conservation of metal binding sites (the Mn^2+^_B_ site of arginases is conserved as the Zn^2+^ site of HDACs) suggest that HDACs and arginases divergently evolved from a common primordial ancestor. As one biological function of arginase is to provide ornithine for polyamine biosynthesis, it is striking that the arginase-deacetylase fold is also recruited for a PDAC function in polyamine metabolism. Our phylogenetic analysis (Fig. 6) indicated that the closest relationship between the HDAC and arginase families is between the ΨDAC domain of *Cavia porcellus* HDAC10 and *H. sapiens* agmatinase (sequence identity=19%). Interestingly, this analysis also suggested that the evolution of PDAC activity in vertebrate HDAC10 and the bacterial deacetylase APAH occurred convergently.

Recently, it has been demonstrated that HDAC10 protects cancer cells from chemotherapeutic drugs by mediating auto-phagy, a survival response to the cellular damage and metabolic stress induced by cytotoxic drugs; indeed, the upregulation of HDAC10 is a marker of poor outcome for advanced stage neuroblastoma patients^32^. However, the knockdown or inhibition of HDAC10 blocks autophagy in a panel of neuroblastoma cells lines, thereby sensitizing these highly malignant cells to the cytotoxic drug doxorubicin^32^. As the suppression of autophagy to sustain the cytotoxicity of chemotherapeutic drugs is a novel strategy for cancer chemotherapy^34^,^35^, HDAC10 is an emerging target for the treatment of advanced-stage neuroblastoma^32^.

The polyamine spermidine is also a key factor in autophagy and increased levels of endogenous or exogenous spermidine induce autophagy and extend lifespan in a variety of cell types, including human immune cells^36^,^37^. Recent studies show that the inhibition of ornithine decarboxylase, which utilizes arginase-derived ornithine to generate putrescine, reduces cellular polyamine levels and suppresses autophagy in eukaryotic cells, thereby attenuating infection by *Trypanosoma cruzi*^38^. Thus, polyamine metabolism is closely linked to autophagy.

Given that HDAC10 is a PDAC with catalytic specificity for the hydrolysis of *N*^8^-acetylspermidine to yield spermidine and acetate, it is possible that the functions of HDAC10 and spermidine in autophagy are linked. With the crystal structure of HDAC10 now in hand, the structure-based design of isozyme-specific inhibitors promises to open new avenues in studying the chemical biology of autophagy as well as the treatment of advanced-stage cancers in which this PDAC is implicated.

## Methods

### General

No statistical methods were used to predetermine sample size. PfuUltra High-Fidelity DNA polymerase (Agilent Technologies) was used for PCR analysis. Restriction enzymes (New England Biolabs) were used according to the manufacturer's specifications. Primers were synthesized by Integrated DNA Technologies. *Escherichia coli* strain NEB5α (New England Biolabs) was used for cloning procedures. Peptides were synthesized by Genscript. *N*-(3-aminopropyl)acetamide was purchased from Chem-Implex International, Inc. Acetylcadaverine was purchased from TCI. *N*^1^-acetylspermidine dihydrochloride was purchased from Cayman Chemicals, Inc. Acetylputrescine, *N*^8^-acetylspermidine dihydrochloride, *N*^1^-acetylspermine trihydrochloride, *N*-butylacetamide and *N*-(aminooctyl)acetamide were purchased form Sigma-Aldrich. *N*^1^,*N*^8^-diacetylspermidine was purchased from Toronto Research Chemicals. Inhibitors trichostatin A (TSA) and suberoylanilide hydroxamic acid (SAHA) were purchased from ApexBio. All substrates and ligands are used without further purification (>95% purity). The transition state analogue inhibitor AAT was synthesized as previously described^24^.

### Protein expression and purification

The human *HDAC10* gene (Q969S8, residues 2–669) and zebrafish *HDAC10* gene (Uniprot Q803K0, residues 2–675) were synthesized and codon-optimized by Genscript (Supplementary Table 2 and Supplementary Table 3, respectively) and then sub-cloned into a modified pET28a(+) vector (a gift from Dr Scott Gradia, University of California, Berkeley; Addgene plasmid 29656) in-frame with a TEV-cleavable N-terminal His-MBP-tag (MBP, maltose binding protein) using ligation independent cloning (PCR primers are listed in Supplementary Table 4). All proteins were expressed in *E. coli* BL21 (DE3) (Agilent) cells in 2x YT medium in the presence of 50 mg l^−1^ kanamycin and purified based on the protocol previously described for HDAC6 (ref. 17). Briefly, expression was induced when OD_600_ reached 1.0 by addition of 75 μM isopropyl β-D-1-thiogalactopyranoside (Carbosynth) and 500 μM ZnSO_4_ (Fisher Scientific), and cell cultures were grown for an additional 18 h at 16 °C. Cells were harvested by centrifugation at 6,000 *g* and resuspended in lysis buffer (50 mM K_2_HPO_4_ (pH 8.0), 300 mM NaCl, 10 mM MgCl_2_, 10% glycerol, 0.1 mg ml^−1^ lysozyme (Sigma), 50 μg ml^−1^ DNase I (Sigma), and protease inhibitor tablets (Roche Applied Science)). Cells were then lysed by sonication and cleared by centrifugation at 26,000 *g* for 1 h at 4 °C. The cleared lysate was loaded onto a Ni-NTA affinity column (Qiagen) and eluted with buffer A (50 mM K_2_HPO_4_ (pH 8.0), 1 mM TCEP, 300 mM NaCl, 300 mM imidazole, 5% glycerol). Protein-containing fractions were loaded onto an Amylose resin column (New England Biolabs).

For human HDAC10, the MBP-tag was not cleaved off. The MBP-fusion hHDAC10 was eluted from Amylose resin by buffer B (20 mM Tris (pH 8.0), 100 mM NaCl, 1 mM TCEP, 5% glycerol) plus 10 mM maltose (Sigma-Aldrich) and further purified by anion-exchange chromatography. For zebrafish HDAC10, the proteins were digested on-column using TEV protease in buffer B to remove the His-MBP tag and further purified by anion-exchange chromatography (HiTrap Q, GE Healthcare). Both zHDAC10 and hHDAC10 were finally loaded onto a HiLoad Superdex 200 column equilibrated in buffer C (50 mM HEPES (pH 7.5), 300 mM KCl, 1 mM TCEP, 5% glycerol). Proteins were concentrated to >10 mg ml^−1^ and flash-cooled in liquid nitrogen and stored at −80 °C for further use.

For the preparation of zHDAC10Δ, 10 mg ml^−1^ of zHDAC10 in buffer C was incubated with trypsin (Sigma-Aldrich) with a 1,000:1 molar ratio of zHDAC10:trypsin overnight at 4 °C and then reloaded onto a HiLoad Superdex 200 column equilibrated in buffer C. Peak fractions corresponding to the co-elution of the PDAC-ΨDAC complex were pooled and concentrated to 30 mg ml^−1^ (the PDAC-ΨDAC domains did not dissociate upon proteolytic nicking of the interdomain linker). The PDAC domain (residues 2–370) remained intact after proteolytic nicking as determined by LC–MS/MS. zHDAC10Δ proteins were flash-cooled in liquid nitrogen and stored at −80 °C for further use.

Single-point mutants were generated using the Quickchange kit (Stratagene); all primers used for PCR mutagenesis are listed in Supplementary Table 4. The zHDAC10 ΔηA2 mutant was prepared by by swapping the helix ηA2 region (D^24^PACEI^29^) with a tetrapeptide segment (S^461^HHP^464^) from the corresponding zHDAC6 CD2 L1 loop. All mutants were expressed and purified using the same procedure as described above for wild-type zHDAC10. Y307F zHDAC10Δ was prepared in the same manner as described above for zHDAC10Δ.

Human HDAC6 CD12, zebrafish HDAC6 CD1 and CD2, and APAH were expressed, purified and assayed as previously reported^14^,^17^.

### Crystallization

The Y307F zHDAC10Δ–AAT complex was crystallized using the sitting drop vapour diffusion method at 4 °C. Protein (30 mg ml^−1^) was first incubated with 5 mM AAT ligand in buffer C on ice for 30 min and then 0.4 μl of protein solution was mixed with 0.4 μl of precipitant solution (0.2 M KH_2_PO_4_, 20% PEG 3350) and equilibrated against an 80 μl reservoir of precipitant solution. Rod-like crystals appeared after 24 h. Crystals were soaked in a cryoprotectant solution consisting of precipitant solution supplemented with 40% ethylene glycol before flash-cooling in liquid nitrogen.

### Crystal structure determination

X-ray diffraction data were recorded at the Stanford Synchrotron Radiation Lightsource, beamline 14-1. Data reduction and integration was achieved with X-ray Detector Software^39^; data collection and reduction statistics are recorded in Table 1. The structure of zHDAC10Δ (Y307F)–AAT complex was solved by molecular replacement using the programme Phaser^40^ and a model of the zHDAC6 CD1-TSA complex (PDB entry 5EEF)^17^ less ligands was used as the search probe. Initial phases from the Phaser solution were judged to be correct based on the initial electron density map, in which much of the omitted ΨDAC domain was clearly visible. The programme phenix.autobuild^41^ was used to generate a ∼70% complete model. Iterative model building was then performed with Coot and model refinement was performed by Phenix^41^,^42^. Residues omitted from the final model due to disorder included S365-A411 (the nicked interdomain loop) and G435, D436 and H454 in the ΨDAC domain. Alternate conformers were observed for R41, N93, C550 and R612 and included in the final model. Nonprotein ligands included in the final model include AAT, phosphate ions and PEG fragment 1PE. Final refinement statistics are recorded in Table 1. The quality of each model was verified with PROCHECK and MolProbity^43^,^44^. Ramachandran statistics showed that all residues adopted allowed conformations. Figures were prepared with Pymol.

### Activity assays

Fluorogenic substrates were custom synthesized by Genscript. Assays were performed at room temperature in triplicate. Briefly, enzymes and substrates were diluted in assay buffer (50 mM Tris-HCl (pH 8.0), 137 mM NaCl, 2.7 mM KCl, 1.0 mM MgCl_2_) at various concentrations. Reactions were initiated by the addition of 25 μl substrate solution to 25 μL enzyme solution (5 μM). Reactions were quenched by adding developer solution (1 μM trypsin and 10 μM SAHA in assay buffer) and allowed to sit for 20 min at room temperature. The reaction mixture was subsequently transferred to a NUNC 384-well optical bottom black plate (Thermo Fisher Scientific) and fluorescence was measured using a Tecan Infinite M1000Pro plate reader (*λ*_ex_=360 nm and *λ*_em_=460 nm). A standard curve using *Fluor de lys* deacetylated standard (Enzo Life Sciences) was used to calculate enzyme activity.

To study steady-state kinetics with non-fluorogenic peptide substrates and acetylpolyamines, a discontinuous LC–MS was employed based on the protocol of assaying nonfluorogenic acetyllysine peptide substrates for HDAC6 (ref. 17). Assays were performed in triplicate. Briefly, 5 μl of enzyme (0.050–20 μM) in HEPES buffer (20 mM HEPES (pH 7.5), 100 mM NaCl, 5 mM KCl, 1 mM MgCl_2_) was added to 35 μl of substrate solution to initiate the reaction. After incubation for 15–60 min at room temperature, the reaction was quenched by addition of 50 μl of 20 mM dansyl chloride dissolved in acetonitrile followed by 10 μl of NaHCO_3_ (1.6 M, pH 10.0). The deacetylation products were derivatized with dansyl chloride at 50 °C for 2 hrs. The deacetylation products were detected by LC–MS using a Waters SQD equipped with an Acquity UPLC (Waters, Milford, MA, USA) and quantified by using the standard curves generated from the mass signals of the corresponding deacetylated polyamines and peptides. Inhibition of HDAC10 activity was measured by using 0.49 μM zHDAC10 with 192 μM *N*^8^-acetylspermidine. Data were analysed by logistic regression for IC_50_ determination and the inhibition constant *K*_i_ was calculated based on the Cheng-Prusoff equation, *K*_i_=IC_50_ / (1+[S]/*K*_M_)^45^. Assays were performed in triplicate at room temperature.

### Electrostatic surface potential and residue conservation

The electrostatic potential of the protein surface was calculated using the Adaptive Poisson-Boltzmann Solver implemented in PyMol^46^. The following parameters were used for calculations: *T*=298.15 K, pH 7.5, AMBER forcefield, linearized Poisson–Boltzmann equation. The PQR file was generated using the web server PDB2PQR (http://nbcr-222.ucsd.edu/pdb2pqr_2.0.0/)^47^. Metal ions were manually added into the PQR file before the electrostatic potential calculation. The charge and radius of metal ions were set to +2 and 0.74 Å for Zn^2+^; +1 and 1.33 Å for K^+^. Residue conservation of 250 HDAC10 orthologues (National Center for Biotechnology Information (NCBI) Protein Reference Sequences) was analysed by ConSurf^48^ (http://consurf.tau.ac.il) and displayed in Pymol.

### Phylogenetic tree

Protein sequences and their annotations were retrieved from the UniProt database^49^; some unannotated sequences were obtained from the NCBI. An unrooted phylogenetic tree of the arginase-deacetylase family was generated based on sequence alignments calculated with ClustalOmega^50^,^51^ and visualized using publicly available tools at the Interactive Tree of Life to generate Fig. 6 (refs 52, 53, 54).

### Model of the zHDAC10–substrate complex

A model of the zHDAC10–substrate complex was prepared by superimposing the crystal structure of the zHDAC6 CD2–RGK(ac)-AMC complex (PDB 5EFN) on the crystal structure of the closely related deacetylase domain of zHDAC10. To generate Fig. 4a, atoms of zHDAC6 CD2 were omitted for clarity and the molecular surface of zHDAC10 was calculated with Pymol. The E274L mutation was then introduced using Coot and an energetically favourable leucine side chain rotamer was selected that coincided with the side chain torsion angles of the original glutamate side chain. The molecular surface was then calculated with Pymol to generate Fig. 4b.

### Data availability

The coordinates and structure factors of the zHDAC10–AAT complex have been deposited in the Protein Data Bank under the accession code 5TD7. The PDB accession codes 3Q9C, 4ZUM, 5EEF, 5EFH, 5EFN, 5G0J and 5IKK were used in this study. The UniProt accession codes Q13547, Q92769, O15379, P56524, Q9UQL6, Q9UBN7, Q8WUI4, Q9BY41, Q9UKV0, Q969S8, Q96DB2, Q803K0, Q6P3E7, H2QLX7, F1RXT2, Q0V897, W5QEU5, Q56970, O25949, Q8I384, Q6TUJ5, Q6WVP6, P05089, P07824, P78540, O08701, P53608, W6KQ49, A0A0M9G714, E9AW07, A4I044, C9ZUY0, V5BBD2, U5KM34, S9WK07, S9W460, A0A0X1KW10, Q4DSA0, L8AVM3, D7ZM62, Q9I6K2, Q9BSE5, Q9I3S3, B5GLC8, Q02959, G8JXJ5, C5DY72, H2AZD2, J8PYB6, Q3JUN4, Q48935, V6AHW3, A0A0J7JFD7, A0A1A9G5G6, B2JF16, Q70I53, A0A0A0D852, X5DVC2, H5ULX3, K0C7Y6, J0BA65, A0A171KNI5, P56523, P53973, O59702, P32561, Q12214, P53096, O67135, P64375, P39607, E1VY17, A0A0I0JU77, A0A0M2PZ25, L8N0Z8, Q6GMJ4, G1KL76, A7E372, Q8BGC1, Q49AR2, Q28H30, Q7SZF1, Q569T0, A0A1D5PN47, C9J8B8, H0VS04, and Q803K0 were used in this study. The NCBI accession codes WP_030688343.1, WP_018062780.1, WP_028738655.1, WP_040747046.1, XP_013837465.1 and XP_016413245.1 were used in this study. All other data are available from the corresponding authors upon reasonable request.

## Additional information

**How to cite this article:** Hai, Y. *et al*. Histone deacetylase 10 structure and molecular function as a polyamine deacetylase. *Nat. Commun.* **8,** 15368 doi: 10.1038/ncomms15368 (2017).

**Publisher's note**: Springer Nature remains neutral with regard to jurisdictional claims in published maps and institutional affiliations.

## Supplementary Material

Supplementary Information Supplementary figures and supplementary tables.

## Figures and Tables

**Table 1:** Data collection and refinement statistics.

	**Y307F zHDAC10Δ–AAT complex**
*Data collection*	
Space group	*P*3_1_21
Cell dimensions	
*a*, *b*, *c* (Å),	80.45, 80.45, 243.04
*α*, *β*, *γ* (^o^)	90.0, 90.0, 120.0
Resolution (Å)	45.82–2.85
	(2.98–2.85)*
*R*_merge_	0.119 (0.930)
*I*/σ*I*	12.9 (2.2)
Completeness (%)	99.0 (100.0)
Redundancy	7.1 (7.3)
CC_1/2_	0.997 (0.682)
	
*Refinement*	
Resolution (Å)	45.82–2.85
	(2.98–2.85)*
No. reflections	22,127/1,092
*R*_work_/*R*_free_	0.181/0.227
	(0.270/0.348)
No. atoms	
Protein	4,935
Ligand/ion	71
Water	38
*B* factors (Å^2^)	
Protein	67
Ligand/ion	84
Water	52
R.m.s.d.	
Bond lengths (Å)	0.004
Bond angles (^o^)	0.7

X-ray diffraction data were collected from a single crystal.

^*^Values in parentheses are for highest-resolution shell.
