# Supplementary material for: Histone deacetylase 10 structure and molecular function as a polyamine deacetylase
Source: Nat Commun. 2017 May 18;8:15368. doi: 10.1038/ncomms15368 (PMC5454378; doi:10.1038/ncomms15368)
Supplement: Supplementary Information — Supplementary figures and supplementary tables. [file ncomms15368-s1.pdf]

Supplementary Table 1. Steady-state kinetics of deacetylases with acetylpolyamines and acetyllysine substrates

hHDAC10	K_M (μM)	k_{cat} (s^{-1})	k_{cat}/K_M ($M^{-1}s^{-1}$)
acetylcadaverine	110 ± 20	0.14 ± 0.02	(1.3 ± 0.2) x 10^3
acetylputrescine	170 ± 50	0.35 ± 0.04	(2.1 ± 0.3) x 10^3
N^8-acetylspermidine	100 ± 10	0.28 ± 0.02	(2.9 ± 0.2) x 10^3
N^1-acetylspermidine	N.D.[1]	N.D.	24 ± 5
N^1-acetylspermine	180 ± 20	0.011 ± 0.002	60 ± 10
N^1,N^8 –diacetylspermidine[2]	150 ± 90	0.14 ± 0.08	900 ± 300
N-(3-aminopropyl)acetamide	N.A.[3]	N.A.	N.A.
N-butylacetamide	N.D.	N.D.	22 ± 5
N-(8-aminooctyl)acetamide	160 ± 40	0.10 ± 0.02	620 ± 50
TK(ac)PIW	N.A.	N.A.	N.A.
AK(ac)P	N.A.	N.A.	N.A.
GAK(ac)	N.A.	N.A.	N.A.
AK(ac)	N.A.	N.A.	N.A.
AK(ac)A	60 ± 20	0.0006 ± 0.0001	11 ± 3
K(ac)	140 ± 40	0.0092 ± 0.0008	66 ± 5
K(ac)*	N.A.	N.A.	N.A.
K(ac)NL	N.A.	N.A.	N.A.
K(ac)NL*	N.A.	N.A.	N.A.
GAK(ac)NLQ	N.A.	N.A.	N.A.
RGK(ac)G	N.D.	N.D.	91 + 8
RGK(ac)-AMC	110 ± 20	0.008 ± 0.002	70 ± 10
GAK(ac)-AMC	10 ± 1	0.00038 ± 0.00002	38 ± 2
**zHDAC10**	**K_M (μM)**	**k_{cat} (s^{-1})**	**k_{cat}/K_M ($M^{-1}s^{-1}$)**
acetylcadaverine	90 ± 30	0.37 ± 0.04	(3.9 ± 0.3) x 10^3
acetylputrescine	160 ± 50	0.7 ± 0.1	(4.2 ± 0.9) x 10^3
N^8-acetylspermidine	130 ± 20	0.58 ± 0.03	(4.6 ± 0.3) x 10^3
N^1-acetylspermidine	N.D.	N.D.	7 ± 2
N^1-acetylspermine	N.D.	N.D.	17 ± 4
N^1,N^8-diacetylspermidine[2]	180 ± 40	0.48 ± 0.08	(2.7 ± 0.3) x 10^3
N-(3-aminopropyl)acetamide	N.D.	N.D.	13 ± 3
N-butylacetamide	900 ± 200	0.06 ± 0.02	60 ± 20
N-(8-aminooctyl)acetamide	170 ± 40	0.21 ± 0.02	(1.2 ± 0.2) x 10^3
TK(ac)PIW	N.A.	N.A.	N.A.
AK(ac)P	N.A.	N.A.	N.A.
GAK(ac)	N.A.	N.A.	N.A.
AK(ac)	N.A.	N.A.	N.A.
AK(ac)A	N.A.	N.A.	N.A.
K(ac)	80 ± 10	0.013 + 0.003	160 + 20
K(ac)*	N.A.	N.A.	N.A.
K(ac)NL	N.A.	N.A.	N.A.
K(ac)NL*	N.A.	N.A.	N.A.
GAK(ac)NLQ	N.A.	N.A.	N.A.
RGK(ac)G	N.D.	N.D.	26 + 5
RGK(ac)-AMC	66 ± 8	0.0015 ± 0.0001	23 ± 9
GAK(ac)-AMC	22 ± 4	0.00033 ± 0.00002	15 ± 2
**zHDAC10Δ**	**K_M (μM)**	**k_{cat} (s^{-1})**	**k_{cat}/K_M ($M^{-1}s^{-1}$)**
acetylcadaverine	60 ± 10	0.036 ± 0.002	600 ± 80
acetylputrescine	80 ± 30	0.081 ± 0.009	980 ± 90
N^8-acetylspermidine	70 ± 30	0.060 ± 0.007	800 ± 100

N^1-acetylspermidine	N.A.	N.A.	N.A.
N^1-acetylspermine	N.D.	N.D.	6 ± 2
RGK(ac)-AMC	120 ± 30	0.0016 ± 0.00001	12 ± 2
GAK(ac)-AMC	40 ± 20	0.00003 ± 0.00001	1.0 ± 0.5
**zHDAC10 E274L**	**K_M (μM)**	**k_{cat} (s^{-1})**	**k_{cat}/K_M ($M^{-1}s^{-1}$)**
acetylcadaverine	60 ± 20	0.020 ± 0.002	350 ± 70
acetylputrescine	160 ± 40	0.034 ± 0.004	220 ± 30
N^8-acetylspermidine	130 ± 40	0.028 ± 0.003	210 ± 30
N^1-acetylspermidine	N.A.	N.A.	N.A.
N^1-acetylspermine	N.A.	N.A.	N.A.
RGK(ac)-AMC	70 ± 10	0.21 ± 0.01	(3.1 ± 0.2) x 10^3
GAK(ac)-AMC	70 ± 20	0.053 ± 0.008	730 ± 90
**zHDAC10 ΔηA2**	**K_M (μM)**	**k_{cat} (s^{-1})**	**k_{cat}/K_M ($M^{-1}s^{-1}$)**
N^8-acetylspermidine	270 ± 50 (K_M')[4]	0.082 ± 0.007	300 ± 30
RGK(ac)-AMC	60 ± 20	0.021 ± 0.002	360 ± 50
**zHDAC10 N93A**	**K_M (μM)**	**k_{cat} (s^{-1})**	**k_{cat}/K_M ($M^{-1}s^{-1}$)**
acetylcadaverine	50 ± 10	0.051 ± 0.004	940 + 20
acetylputrescine	130 ± 30	0.126 ± 0.008	930 ± 40
N^8-acetylspermidine	150 ± 30	0.123 ± 0.009	800 ± 40
N^1-acetylspermidine	N.A.	N.A.	N.A.
N^1-acetylspermine	N.D.	N.D.	50 ± 10
RGK(ac)-AMC	110 ± 40	0.0013 ± 0.0002	12 ± 1
GAK(ac)-AMC	60 ± 30	0.00007 ± 0.00001	1.0 ± 0.6
**zHDAC10 D94A**	**K_M (μM)**	**k_{cat} (s^{-1})**	**k_{cat}/K_M ($M^{-1}s^{-1}$)**
acetylcadaverine	260 ± 60	0.29 ± 0.04	(1.1 ± 0.2) x 10^3
acetylputrescine	110 ± 10	0.24 ± 0.01	(2.2 ± 0.2) x 10^3
N^8-acetylspermidine	140 ± 20	0.90 ± 0.04	(6.2 ± 0.3) x 10^3
N^1-acetylspermidine	N.A.	N.A.	N.A.
N^1-acetylspermine	N.D.	N.D.	22 ± 4
RGK(ac)-AMC	150 ± 40	0.0013 ± 0.0002	9 ± 1
GAK(ac)-AMC	17 ± 4	0.00046 ± 0.00002	27 ± 3
**APAH**	**K_M (μM)**	**k_{cat} (s^{-1})**	**k_{cat}/K_M ($M^{-1}s^{-1}$)**
acetylcadaverine	22 ± 4	1.5 ± 0.1	(6.8 ± 0.7) x 10^4
acetylputrescine	120 ± 10 (K_M')[5]	1.5 ± 0.1	(1.0 ± 0.1) x 10^4
N^8-acetylspermidine	80 ± 20	2.5 ± 0.3	(3.0 ± 0.5) x 10^4
N^1-acetylspermidine	70 ± 20	0.60 ± 0.05	(7.4 ± 0.6) x 10^3
N^1-acetylspermine	60 ± 10	0.20 ± 0.01	(2.5 ± 0.9) x 10^3
N-(3-aminopropyl)acetamide	180 ± 60	1.3 ± 0.2	(7 ± 1) x 10^3
N-(8-aminooctyl)acetamide	110 ± 20	1.1 ± 0.1	(1.0 ± 0.1) x 10^4
N-butylacetamide	N.D.	N.D.	380 ± 90
K(ac)	220 ± 70	0.13 ± 0.02	600 ± 100
RGK(ac)-AMC	110 ± 10	0.017 ± 0.001	150 ± 20
GAK(ac)-AMC	120 ± 20	0.0067 ± 0.0005	50 ± 5
**hHDAC6 CD12**	**K_M (μM)**	**k_{cat} (s^{-1})**	**k_{cat}/K_M ($M^{-1}s^{-1}$)**
acetylcadaverine	N.D.	N.D.	80 ± 4
acetylputrescine	N.D.	N.D.	18 ± 3
N^8-acetylspermidine	N.D.	N.D.	39 ± 7
N^1-acetylspermidine	N.D.	N.D.	0.6 ± 0.3
N^1-acetylspermine	N.D.	N.D.	1.8 ± 0.3
RGK(ac)-AMC[5]	11 ± 4	0.049 ± 0.009	(4.4 ± 0.8) x 10^3
GAK(ac)-AMC	14 ± 2	0.039 ± 0.007	(2.8 ± 0.4) x 10^3
**zHDAC6 CD1**	**K_M (μM)**	**k_{cat} (s^{-1})**	**k_{cat}/K_M ($M^{-1}s^{-1}$)**
acetylcadaverine	N.D.	N.D.	5 ± 1
acetylputrescine	N.D.	N.D.	0.93 ± 0.04

N^{8}-acetylspermidine	N.D.	N.D.	1.2 ± 0.5
N^{1}-acetylspermidine	N.A.	N.A.	N.A.
N^{1}-acetylspermine	N.A.	N.A.	N.A.
RGK(ac)-AMC [6]	43 ± 6	0.20 ± 0.06	(4.7 ± 0.3) x 10^3
**zHDAC6 CD2**	**K_M (μM)**	**k_{cat} (s^{-1})**	**k_{cat}/K_M ($M^{-1}s^{-1}$)**
acetylcadaverine	N.D.	N.D.	28 ± 2
acetylputrescine	N.D.	N.D.	30 ± 5
N^{8}-acetylspermidine	N.D.	N.D.	29 ± 3
N^{1}-acetylspermidine	N.D.	N.D.	17 ± 2
N^{1}-acetylspermine	N.A.	N.A.	N.A.
N-(3-aminopropyl)acetamide	N.D.	N.D.	2.0 ± 0.5
GAK(ac)NLQ	150 ± 20	1.26 ± 0.07	8.3 x 10^3
RGK(ac)-AMC [6]	22 ± 9	0.69 ± 0.08	(3.1 ± 0.5) x 10^4

Data represent mean ± s.e.m. The molecular structures of all substrates are illustrated in Supplementary Fig. 2. [1]N.D., not determined; individual k_{cat} and K_M values were not determined for enzyme-substrate pairs that did not exhibit saturation kinetics. [2]Substrate inhibition was observed. [3]N.A., not active. [4]Cooperativity was observed with Hill coefficient h = 3.0. [5]Cooperativity was observed with Hill coefficient h = 1.9. [6]Data from ref. 17.

Supplementary Table 2. Sequence of the codon-optimized human HDAC10 gene

AGCAGCGGCACCGCGCTGGTTTATCACGAGGACATGACCGCGACCCGTCTGCTGTGGGATGACCCGGA
ATGCGAGATTGAACGCCCGGAACGTCTGACCGCGGCGCTGGATCGTCTGCGTCAACGTGGCCTGGAACA
ACGTTGCCTGCGTCTGAGCGCGCGTGAGGCGAGCGAGGAAGAGCTGGGTCTGGTGCACAGCCCGGAAT
ACGTGAGCCTGGTTCGTGAGACCCAGGTTCTGGGCAAGGAAGAGCTGCAAGCGCTGAGCGGCCAGTTC
GATGCGATCTATTTTCACCCGAGCACCTTCCACTGCGCGCGTCTGGCGGCGGGTGCGGGTCTGCAACTG
GTGGACGCGGTTCTGACCGGTGCGGTGCAGAACGGTCTGGCGCTGGTTCGTCCGCCGGGTCACCACGG
TCAACGTGCTGCGGCGAACGGCTTCTGCGTGTTTAACAACGTTGCGATTGCTGCGGCGCACGCGAAGCA
GAAACACGGTCTGCACCGTATCCTGGTGGTTGACTGGGATGTGCACCACGGTCAAGGCATTCAGTACCT
GTTTGAAGACGATCCGAGCGTTCTGTACTTCAGCTGGCACCGTTATGAACACGGCCGTTTCTGGCCGTTT
CTGCGTGAAAGCGATGCGGATGCGGTGGGTCGTGGTCAAGGTCTGGGCTTTACCGTGAACCTGCCGTGG
AACCAGGTTGGTATGGGCAACGCGGATTATGTTGCGGCGTTCCTGCACCTGCTGCTGCCGCTGGCGTTC
GAATTTGACCCGGAGCTGGTGCTGGTTAGCGCGGGTTTTGATAGCGCGATCGGCGACCCGGAAGGTCAA
ATGCAAGCGACCCCGGAGTGCTTTGCGCACCTGACCCAGCTGCTGCAAGTGCTGGCGGGTGGCCGTGT
GTGCGCGGTTCTGGAGGGTGGCTACCACCTGGAAAGCCTGGCGGAGAGCGTGTGCATGACCGTTCAAA
CCCTGCTGGGCGATCCGGCGCCGCCGCTGAGCGGTCCGATGGCGCCGTGCCAGAGCGCGCTGGAAAG
CATTCAAAGCGCGCGTGCGGCGCAGGCGCCGCACTGGAAGAGCCTGCAGCAACAGGACGTGACCGCGG
TTCCGATGAGCCCGAGCAGCCACAGCCCGGAGGGTCGTCCGCCGCCGCTGCTGCCGGGTGGCCCGGTT
TGCAAAGCGGCGGCGAGCGCGCCGAGCAGCCTGCTGGACCAGCCGTGCCTGTGCCCGGCGCCGAGCG
TGCGTACCGCGGTTGCGCTGACCACCCCGGATATCACCCTGGTGCTGCCGCCGGACGTTATTCAACAGG
AAGCGAGCGCGCTGCGTGAAGAAACCGAAGCGTGGGCGCGTCCGCACGAGAGCCTGGCGCGTGAAGA
GGCGCTGACCGCGCTGGGCAAGCTGCTGTACCTGCTGGACGGCATGCTGGATGGTCAAGTGAACAGCG
GTATTGCGGCGACCCCGGCGAGCGCTGCGGCGGCGACCCTGGATGTTGCGGTTCGTCGTGGCCTGAGC
CACGGTGCGCAACGTCTGCTGTGCGTTGCGCTGGGTCAGCTGGATCGTCCGCCGGATCTGGCGCATGAT
GGCCGTAGCCTGTGGCTGAACATCCGTGGTAAAGAGGCGGCGGCGCTGAGCATGTTTCACGTGAGCACC
CCGCTGCCGGTTATGACCGGTGGCTTCCTGAGCTGCATTCTGGGCCTGGTGCTGCCGCTGGCGTATGGT
TTTCAACCGGACCTGGTGCTGGTTGCGCTGGGTCCGGGCCACGGTCTGCAAGGTCCGCATGCGGCGCT
GCTGGCGGCGATGCTGCGTGGTCTGGCGGGTGGCCGTGTTCTGGCGCTGCTGGAAGAGAACAGCACCC
CGCAGCTGGCGGGTATTCTGGCGCGTGTGCTGAACGGTGAAGCGCCGCCGAGCCTGGGTCCGAGCAGC
GTTGCGAGCCCGGAGGATGTTCAAGCGCTGATGTATCTGCGTGGTCAACTGGAGCCGCAATGGAAAATG
CTG

Supplementary Table 3. Sequence of the codon-optimized zebrafish HDAC10 gene

GCGAGCGGTAGCGCGCTGATTTTTGATGAGGAGATGAGCCGTTACAAGCTGCTGTGGACCGATCCGGCG
TGCGAGATTGAGGTTCCGGAACGTCTGACCGTGAGCTACGAAGCGCTGCGTACCCATGGTCTGGCGCAG
CGTTGCAAAGCGGTGCCGGTTCGTCAGGCGACCGAGCAAGAAATCCTGCTGGCGCACAGCGAGGAATAC
CTGGAAGCGGTGAAACAAACCCCGGGCATGAACGTTGAGGAACTGATGGCGTTCAGCAAGAAATACAAC
GACGTTTATTTTCACCAGAACATTTATCACTGCGCGAAACTGGCGGCGGGTGCGACCCTGCAACTGGTGG
ATAGCGTTATGAAACGTGAGGTGCGCAACGGTATGGCGCTGGTTCGTCCGCCGGGTCACCACAGCCAGC
GTAGCGCGGCGAACGGTTTCTGCGTGTTTAACAACGTTGCGTTCGCGGCGCTGTACGCGAAGAAAAACT
ATAACCTGAACCGTATCCTGATTGTGGACTGGGATGTTCACCACGGTCAGGGCATCCAATACTGCTTCGA
GGAAGACCCGAGCGTGCTGTACTTTAGCTGGCACCGTTATGAACACCAGAGCTTCTGGCCGAACCTGCC
GGAGAGCGATTATAGCAGCGTGGGCAAGGGCAAAGGTAGCGGCTTTAACATTAACCTGCCGTGGAACAA
GGTTGGCATGACCAACAGCGACTACCTGGCGGCGTTCTTTCACGTGCTGCTGCCGGTTGCGTATGAATTC
GATCCGGAGCTGGTGATCGTTAGCGCGGGTTTTGACAGCGCGATTGGTGATCCGGAGGGCGAAATGTGC
GCGCTGCCGGAAATCTTCGCGCACCTGACCCACCTGCTGATGCCGCTGGCGGCGGGTAAAATGTGCGTG
GTTCTGGAGGGTGGCTACAACCTGACCAGCCTGGGTCAGAGCGTGTGCCAAACCGTGCATAGCCTGCTG
GGCGACCCGACCCCGCGTATCAGCGGTCTGGGCACCGCGTGCGATAGCGCGCTGGAAAGCATTCAGAA
CGTGCGTAACGTTCAAAGCAGCTATTGGAGCAGCTTTAAGCACCTGGCGCAGAGCGAGACCAACCCGAA
ACGTCCGCGTCTGGATGCGACCAACGGTGGCCCGAAGGAGAGCAGCGAACCGGCGAGCGAAAGCAACC
CGAAGAAAACCGCGCAAGATATCGTTTGGCCGGAACCGCTGAAGCGTATGCCGGCGAGCGTGCGTACCG
TGGTGGTGCCGCCGCCGGGTGTGGAACTGACCCTGCCGAAAAACTGCCAGCACAGCGGCGACATCAGC
GAAAGCACCGCGAAAGAGGTGCAGCGTATTCGTGATAAACACTTCCACGACCTGACCGATCAAAACATTC
TGCGTAGCCTGGGCAACATCATTAGCGTGCTGGACCGTATGATGCGTAGCGATGAAGTTTGCAACGGTTG
CGTTGTGGTTAGCGACCTGAGCGTGAGCGTTCAGTGCGCGCTGCAGCATGCGCTGACCGAGCCGGCGG
AACGTGTTCTGGTGGTGTACGTTGGTGACGGCGAGCTGCCGGTGAAGACCAACGATGGCAAAGTGTTTC
TGGTTCAGATCTGCACCAAGGAGACCGAAGATAAATGCGTTAACCGTCTGACCCTGTGCCTGCGTGAGG
GTGAAAGCCTGACCGCGGGCTTCATGCAAGCGCTGCTGGGTCTGATCCTGCCGGTGGCGTACGAATTTA
ACCCGGCGCTGGTGCTGGGCATTGTTGAGGAAACCGCGGCGAAGACCCGTCTGATGCGTGTTTGGGGT
CACATGACCTGCCTGATCCAGGGTCTGGCGCGTGGTCGTATGCTGACCCTGCTGCAAGGTTATGACAAA
GATCTGCTGGAACTGACCGTGAGCGCGCTGAGCGGTGCGAGCATTAGCCCGCTGGGTCCGCTGCGTGC
GCCGAAGCCGGAGGACGTTGAAATGATGGAGAAACAACGCCAACGCCTGCAAGAACGCTGGGGTCTGCT
GCGCTGCACCGTTAGCGAAAGCTGG

Supplementary Table 4. PCR primers for ligation independent cloning and site-directed mutagenesis

Oligonucleotide	Sequence
zHDAC10_LIC_For	5’-*TACTTCCAATCCAATGCAGGTGGCAGTGCA*GCGAGCGGTAGCGCGC-3’
zHDAC10_LIC_Rev	5’-*TTATCCACTTCCAATGTTATTA*CCAGCTTTCGCTAACGGTGCAG-3’
hHDAC10_LIC_For	5’-*TACTTCCAATCCAATGCAGGTGGC*AGCAGCGGCACCGCGCTGGTTTATCACG -3’
hHDAC10_LIC_Rev	5’-*TTATCCACTTCCAATGTTATTA*ATGGGTCTGCAGCATTTTCCATTGCGGCTCCAGTTG-3’
zHDAC10_ΔηA2_For	5’-GCCGTTACAAGCTGCTGTGGGACTCGCATCACCCGGAGGTTCCGGAACGTCTGAC-3’
zHDAC10_ΔηA2_Rev	5’-GTCAGACGTTCCGGAACCTCCGGGTGATGCGAGTCCCACAGCAGCTTGTAACGGC-3’
zHDAC10_D94A_For	5’-GTTCAGCAAGAAATACAACGCAGTTTATTTTCACCAGAACATTTATC-3’
zHDAC10_D94A_Rev	5’-GATAAATGTTCTGGTGAAAATAAACTGCGTTGTATTTCTTGCTGAAC-3’
zHDAC10_N93A_For	5’-GGCGTTCAGCAAGAAATACGCCGACGTTTATTTTCACCAGAACATTTATCACTGCGC-3’
zHDAC10_N93A_Rev	5’-GCGCAGTGATAAATGTTCTGGTGAAAATAAACGTCGGCGTATTTCTTGCTGAACGCC-3’
zHDAC10_E274L_For	5’-GATTGGTGATCCGCTGGGCGAAATGTG-3’
zHDAC10_E274L_Rev	5’-CACATTTCGCCCAGCGGATCACCAATC-3’
zHDAC10_Y307F_For	5’-GGAGGGTGGCTTTAACCTGACCAG-3’
zHDAC10_Y307F_Rev	5’-CTGGTCAGGTTAAAGCCACCCTCC-3’

Italics indicate overhang introduced for ligation-independent cloning (LIC). Altered codons for site-directed mutagenesis and loop swapping are underlined. For = forward, Rev = reverse.

Supplementary Table 5. Acronyms, species, and UniProt accession numbers corresponding to the phylogenetic tree in Fig. 6.

Arginases (9)
HpARG, *Helicobacter pylori*, Arginase, O25949
PfARG, *Plasmodium falciparum*, Arginase, Q8I384
LmARG, *Leishmania mexicana*, Arginase, Q6TUJ5
SmARG, *Schistosoma mansoni*, Arginase, Q6WVP6
HsARG1, *Homo sapiens,* Arginase 1, P05089
RnARG1, *Rattus norvegicus*, Arginase 1, P07824
HsARG2, *Homo sapiens,* Arginase 2, P78540
RnARG2, *Rattus norvegicus,* Arginase 2, O08701
BcARG, *Bacillus caldovelox,* Arginase, P53608

Pseudo-arginases (ΨArg), lacking a metal-binding site (9)
PhArg, *Phytomonas sp.* EM1, Unnamed Protein Arginase/Deacetylase Superfamily, W6KQ49
LpArg, *Leptomonas pyrrhocoris,* Putative Agmatinase/Arginase, A0A0M9G714
LmArg, *Leishmania mexicana,* Putative Agmatinase/Arginase, E9AW07
LiArg, *Leishmania infantum,* Putative Agmatinase/Arginase, A4I044
TbArg, *Trypanosoma bruci,* Putative Agmatinase/Arginase, C9ZUY0
TcArg, *Trypanosoma cruzi,* Putative Agmatinase/Arginase, V5BBD2
HmArg, *Herpetomonas muscarum,* Putative Agmatinase/Arginase, U5KM34
AdArg, *Angomonas deanei,* Putative Agmatinase/Arginase, S9WK07
ScArg, *Strigomonas culicis,* Putative Agmatinase/Arginase, S9W460

Formiminoglutamases (FIGase) and ureohydrolases (9)
VcFIGase, *Vibrio cholerae,* FIGase, A0A0X1KW10
TcFIGase, *Trypanosoma cruzi,* FIGase/ Putative Arginase, Q4DSA0
BcFIGase, *Bacillus subtilis,* Formiminoglutamate Hydrolase, L8AVM3
SmDstH, *Streptomyces* species NRRL B-1347, Agmatinase, WP_030688343.1*
EcAGMAT, *Escherichia coli,* Agmatinase, D7ZM62
PaGpuA, *Pseudomonas aeruginosa,* Guanidinopropionase, Q9I6K2
HsAGMAT, *Homo sapiens,* Agmatinase, Q9BSE5
PaGbuA, *Pseudomonas aeruginosa,* Guanidinobutyrase, Q9I3S3
ScPAH, *Streptomyces clavuligerus,* Proclavaminate Amidohydrolase, B5GLC8

Yeast Hos3 homologues (5)
ScHos3, *Saccharomyces cerevisiae,* Hos3, Q02959
EcHos3, *Eremothecium cymbalariae,* Hos3, G8JXJ5
ZrHos3, *Zygosaccharomyces rouxii,* Hos3, C5DY72
KaHos3, *Kazachstania africana,* Hos3, H2AZD2
SaHos3, *Saccharomyces arboricola,* Hos3, J8PYB6

Bacterial acetylpolyamine amidohydrolases (APAH) (7)
BpAPAH, *Burkholderia pseudomalli,* APAH, Q3JUN4
MrAPAH, *Mycoplana ramosa* APAH, Q48935
PaAPAH, *Pseudomonas aerugenosa,* APAH, V6AHW3
MsAPAH, *Marinobacter subterrani,* APAH, A0A0J7JFD7**
MmAPAH, *Martelella mediterranea,* APAH, WP_018062780.1*
RsAPAH, *Rhizobium selenitireducens,* APAH, WP_028738655.1*
ShinellaAPAH, *Shinella* species HZN7, APAH, A0A1A9G5G6

Bacterial histone deacetylase-like amidohydrolases (HDAH) (9)
BpHDAH, *Burkholderia phymatum,* HDAH, B2JF16
AsHDAH, *Alcaligenaceae* species, HDAH, Q70I53
ILAcuC, *Inquilinus limosus,* HDAH, A0A0A0D852
CgAcuC, *Corynebacterium glyciniphilum,* HDAH, X5DVC2
GtHDAH, *Gordonia terrae,* HDAH, H5ULX3
NtAcuC, *Nocardia transvalensis,* HDAH, WP_040747046.1*
AdHDAH, *Alcanivorax dieselolei,* HDAH, K0C7Y6
AfHDAH, *Alcaligenes faecalis,* HDAH, J0BA65
KgAcuC, *Kerstersia gyiorum,* HDAH, A0A171KNI5

Class II HDACs (9)
HsHDAC10, *Homo sapiens,* HDAC10, Q969S8
HsHDAC6CD1, *Homo sapiens,* HDAC6 Catalytic Domain 1, Q9UBN7
HsHDAC6CD2, *Homo sapiens,* HDAC6 Catalytic Domain 2, Q9UBN7
HsHDAC7, *Homo sapiens,* HDAC7, Q8WUI4
HsHDAC5, *Homo sapiens,* HDAC5, Q9UQL6
HsHDAC4, *Homo sapiens,* HDAC4, P56524
HsHDAC9, *Homo sapiens,* HDAC9, Q9UKV0
SpClr3, *Schizosaccharomyces pombe,* Clr3, P56523
ScHda1, *Saccharomyces cerevisiae,* Hda1, P53973

Class I HDACs (9)
HsHDAC3, *Homo sapiens,* HDAC3, O15379
HsHDAC2, *Homo sapiens,* HDAC2, Q92769
HsHDAC1, *Homo sapiens,* HDAC1, Q13547
SpClr6, *Schizosaccharomyces pombe,* Clr6, O59702
ScRpd3, *Saccharomyces cerevisiae,* Rpd3, P32561
ScHos1, *Saccharomyces cerevisiae,* HOS1, Q12214
ScHos2, *Saccharomyces cerevisiae,* Hos2, P53096
HsHDAC8, *Homo sapiens,* HDAC8, Q9BY41
AaHDLP, *Aquifex aeolicus,* Histone Deacetylase-Like Protein, O67135

Bacterial acetoin utilization proteins (AcuC) (4)
SaAcuC, *Staphylococcus aureus,* Acetoin Utilization Protein AcuC, P64375
BsAcuC, *Bacillus subtilis,* acetoin Utilization Protein AcuC, P39607
GaAcuC, *Glutamicibacter arilaitensis,* Acetoin Utilization Protein AcuC, E1VY17
StreptaAcuC, *Streptomyces acidiscabies,* Acetoin Utilization Protein AcuC, A0A0I0JU77

Class IV HDACs (7)
HsHDAC11, *Homo sapiens*, HDAC11, Q96DB2
PhHDAC11, *Prochlorothrix hollandica,* HDAC, A0A0M2PZ25
PbHDAC11, *Pseudanabaena biceps,* HDAC, L8N0Z8
DrHDAC11, *Danio rerio,* HDAC11, Q6GMJ4
AcHDAC11, *Anolis carolinensis,* HDAC11, G1KL76
BsHDAC11, *Bos taurus,* HDAC11, A7E372
SsHDAC11, *Sus scrofa,* HDAC11, XP_013837465.1*

Uncharacterized Protein Family UPF0489 (4)
MmUPF0489, *Mus musculus,* UPF0489, Q8BGC1
HsUPF0489, *Homo sapiens,* UPF0489, Q49AR2
XtUPF0489, *Xenopus tropicalis,* UPF0489, Q28H30
DrUPF0489, *Danio rerio,* UPF0489, Q7SZF1

Pseudo-deacetylases (ΨDAC), lacking a metal binding site (6)
XlPseudoDAC, *Xenopus laevis,* HDAC10, Q569T0
GgPseudoDAC, *Gallus gallus,* HDAC10, A0A1D5PN47
HsPseudoDAC, *Homo sapiens,* HDAC10, C9J8B8
CpPseudoDAC, *Cavia porcellus,* HDAC10, H0VS04
DrPseudoDAC, *Danio rerio,* HDAC10, Q803K0
SrPseudoDAC, *Sinocyclocheilus rhinocerous,* HDAC10, XP_016413245.1*

*Sequence not annotated in the UniProt database, accession code denotes the NCBI sequence ID
**Annotated as an acetoin utilization protein (AcuC) in the UniProt database

```
                       10        20        30        40        50        60        70        80        90
D_rerio       MASGSALIFDEEMSRYKLLWTDPACEIEVPERLTVSYEALRTHGLAQRCKAVPVRQATEQEILLAHSEEYLEAVKQTPGMNVEELMAFSKKY
H_sapiens     --MGTALVYHEDMTATRLLWDDPECEIERPERLTAALDRLRQRGLEQRCLRLSAREASEEELGLVHSPEYVSLVRETQVLGKEELQALSGQF
M_musculus    --MGTALVYHEDMTATRLLWDDPECEIECPERLTAALDGLRQRGLEERCLCLSACEASEEELGLVHSPEYIALVQKTQTLDKEELHALSKQY
P_troglodytes --MGTALVYHEDMTATRLLWDDPECEIERPERLTAALDRLRQRGLEQRCLRLSAREASEEELGLVHSPEYVSLVRETQVLGKEELQALSGQF
S_scrofa      --MGTALVYHEDMTAARLLWDDPECEIERPERLTTALERLRQRGLEQRCLRLVAREASEAELGLVHSPEYVALLRGTQALSTEELQALSRQF
B_taurus      --MGTALVYHEDMTATRLLWDDPECEIECPERLTTALERLQQHGLKQRCLQLVAREASEAELGLVHSPEYVALLQGTQALGTRELQALSKEY
O_aries       --MGTALVYHEDMTAARLLWDDPECEIECPERLTTALERLQQRGLEQRCLRLVAREASEAELGLVHSPEYVALLRGTQALGTRELQALSKQY
X_laevis      MACGTALVYDEEMMSYKLLWDDPECSIEVPERLSSSYKRLQDYDLVKRCIQLPVREATDEEITLVHSHDYLQVVKSTQTMNEKELKEISQKY

                     100       110       120       130       140       150       160       170       180
D_rerio       NDVYFHQNIYHCAKLAAGATLQLVDSVMKREVRNGMALVRPPGHHSQRSAANGFCVFNNVAFAALYAKKNYNLNRILIVDWDVHHGQGIQYC
H_sapiens     DAIYFHPSTFHCARLAAGAGLQLVDAVLTGAVQNGLALVRPPGHHGQRAAANGFCVFNNVAIAAAHAKQKHGLHRILVVDWDVHHGQGIQYL
M_musculus    NAVYFHPDTFHCARLAAGAALQLVDAVLTGAVHNGLALVRPPGHHSQRAAANGFCVFNNVALAAKHAKQKYGLQRILIVDWDVHHGQGIQYI
P_troglodytes DAIYFHPSTFHCARLAAGAGLQLVDAVLTGAVQNGLALVRPPGHHSQRAAANGFCVFNNVAIAAAHAKQKHGLHRILVVDWDVHHGQGIQYL
S_scrofa      DAVYFHPSTFHCARLAAGAALQLVDAVMAGVVRNGLALVRPPGHHSQRAAANGFCVFNSVAIAAKHAQQKHGLHRILIVDWDIHHGQGTQYI
B_taurus      DAVYLHPSTFHCARLAVGAALQLVDAVLTGAVRNGLALVRPPGHHSQRATANGFCVFNNVAIAAKHAQQKHGLRRILIVDWDVHHGQGIQYI
O_aries       DAVYLHPSTFHCARLAAGAALQLVDAVLTGAVRNGLALVRPPGHHSQRATANGFCVFNNVAIAAKHAQQKHGLHRILIVDWDIHHGQGIQYI
X_laevis      TAVFYHQNSFRCAKLSLGGTLQLVDAILTREVQNGMAIVRPPGHHSQRNQGNGFCVFNNVAIAAEYAKKKYKLERILIVDWDVHHGQGIQYI

                   190       200       210       220       230       240       250       260       270
D_rerio       FEEDPSVLYFSWHRYEHQSFWPNLPESDYSSVGKGKGSGFNINLPWNKVGMTNSDYLAAFFHVLLPVAYEFDPELVIVSAGFDSAIGDPEGE
H_sapiens     FEDDPSVLYFSWHRYEHGRFWPFLRESDADAVGRGQGLGFTVNLPWNQVGMGNADYVAAFLHLLLPLAFEFDPELVLVSAGFDSAIGDPEGQ
M_musculus    FNDDPSVLYFSWHRYEHGSFWPFLPESDADAVGQGQGQGFTVNLPWNQVGMGNADYLAAFLHVLLPLAFEFDPELVLVSAGFDSAIGDPEGQ
P_troglodytes FEDDPSVLYFSWHRYEHGRFWPFLRESDADAVGRGQGLGFTVNLPWNQVGMGNADYVAAFLHLLLPLAFEFDPELVLVSAGFDSAIGDPEGQ
S_scrofa      FEDDPSVLYFSWHRYEHGRFWPYLRESDADTVGRGRGLGFTVNLPWNQVGMGNADYMAAFLQVLLPLAFEFNAELVLVSAGFDSAIGDSEGQ
B_taurus      FEDDPSVLYFSWHRYEHGHFWPCLRESDADAVGRGRGLGFTVNLPWNQVGMGNADYVAAFLHVLLPLAFEFDPELVLVSAGFDSAIGDPEGQ
O_aries       FEDDPSVLYFSWHRYEHGHFWPCLRESDADAVGRGRGLGFTVNLPWNQVGMGNADYVAAFLHVLLPLAFEFDPELVLVSAGFDSAIGDPEGQ
X_laevis      FEEDPSVLYFSWHRYEHKTFWPYLRESDYDVIGRGKGTGFNINLPWNKVGMGNADYIAAFFHVLLPLAFEFNPELVLVSAGYDSAIGDPEGR

                 280       290       300       310       320       330       340       350       360
D_rerio       MCALPEIFAHLTHLLMPLAAGKMCVVLEGGYNLTSLGQSVCQTVHSLLGDPTPRISGLGTACDSALESIQNVRNVQSSYWSSFKHLAQSETN
H_sapiens     MQATPECFAHLTQLLQVLAGGRVCAVLEGGYHLESLAESVCMTVQTLLGDPAPPLSGPMAPCQSALESIQSARAAQAPHWKSLQQQDVTAVP
M_musculus    MQATPECFAHLTQLLQVLAGGRICAVLEGGYHLESLAQSVCMMVQTLLGDPTPPLLGLMVPCQSALESIQSVQTAQTPYWTSLQQN-VAPV-
P_troglodytes MQATPECFAHLTQLLQVLAGGRVCAVLEGGYHLESLAESVCMTVQTLLGDPAPPLSGPMVPCQSALESIQSARAAQAPHWKSLQQQDVTAVP
S_scrofa      MQATPECFAHLTQLLQVLAGGRVCAVLEGGYHLESLSQSVCMMVQALLGDPAPPLSGPMVPHGSALQSIQSVRAAQAPHWMSLRQQGVAPV-
B_taurus      MLATPECFAHLTHLLQVLAGGRVCAVLEGGYHLESLSQSVCMMVRALLGDPALPLSGPMEPHGSALESLQCVRAAQAPHWVSLQQQGAAPV-
O_aries       MLATPECFAHLTQLLQVLKGEGAWPEAGGGYHLESLSQSVCMMVQALLGDPALPLSGPMEPHGSALESLQCVRAAQAPHWVSLRQQGAAPV-
X_laevis      MCATPECFSHLTNMLMNLAGGKLCAVLEGGYNLRSLAESVCMTVRTLLGDPLPRLTGEMTPCHSALESIQNVRAAHTPYWKCLLYNEIKSAH

               370       380       390       400       410       420       430       440       450
D_rerio       PKRPRLDATNGGPKESSEPASESNPKKTAQD---IVW-----PEPLKRMPASVRTVV-V-PPPGVELTLPKNCQHSGDISESTAKEVQRI-R
H_sapiens     -----MSPSSHSPEGRPPPLLPGGPVCKAAA----SAPSSLLDQPCLCPAPSVRTAVALTTPDI-TLVLPPDVIQQEASA--LREETEAW--
M_musculus    -----LSSSTHSPEERSLRLLGESPTCAVAE----DSLSPLLDQLCLRPAPPICTAVASTVPGA-ALCLPPGVLHQEGSV--LREETEAW--
P_troglodytes -----MSPSSHSPEGRPPPLLPGGPVCKAAA----SALSSLLDQPCLCPAPSVRTAVALTTPDI-TSVLPPDVIQQEASA--LREETEAW--
S_scrofa      -----LSSSTRSPGQGPSPPMPAGPEFKTAAAQAVAALSSLLDQLRLRPTPPVRTALALPAPLDGDLVLPHDVLCQEGSA--PQEETQAW--
B_taurus      -----LSPGTPCPEGRPSPLPLGEPQFKAVVTQAAAALSSLLDQLRLHPTPPVRVAVALIAPDTG-LALPPGVLCEEGSL--PQEETQAW--
O_aries       -----LSSGTRCPEGRPSPLPPGEPEFKAVVTQAAAALSSLLDQLRLHPTPPVRVAVALTAPVTG-LALPPSVLCEEGSL--PQGEGLGQ--
X_laevis      -----DPSS----EGDSQHSSDQQQNFDTAMFD--IFLDSHMKKI-EFAVPPLRTCAVL--PEGCCLALPDGVLVEEKTA--TREHIIACSS

                       470       480       490       500       510       520       530       540       550
D_rerio       DKHFHDLTDQNILRSLGNIISVLDRMMRSDEVCNGCVVVSD--LSVSVQCALQHALTEPAERVLVVYVGDGELP-VKTNDGKVFLVQICTKE
H_sapiens     ARPHESLAREEALTALGKLLYLLDGMLDGQV-NSGIAATPASAAAATLDVAVRRGLSHGAQRLLCVALGQLDRPPDLAHDGRSLWLNIRGKE
M_musculus    ARLHKSRFQDEDLATLGKILCLLDGIMDGQI-RNAIATTTALATAATLDVLIQRCLARRAQRVLCVALGQLDRPLDLADDGRILWLNIRGKD
P_troglodytes ARPHESLAREEALTALGKLLYLLDGMLDGQV-NSGIAATPASAAAATLDVAVRRGLSHGAQRLLCVALGQLDRPPDLAHDGRSLWLNIRGKE
S_scrofa      ARPHEALAQDKALSALGRVLHLLDRILDGQV-SSGIAATPGPAVAATLDVAIRCGLSHGAQRLLCVAVGQLDRPPHLTDDGDCGYVCPPEI-
B_taurus      ARPHEALAQDGALTALGKVLYLLDRILDGQV-SSGMAATPVPAAAATLDVAVRYGLSHGAQRLLCVAVGQLDRPPGLTDDGRNLWLNIGGEE
O_aries       HRPHEALAQDEALIALGKVLYLLDRILDGQV-SSGTAATPVPAAAATLDVAVRYGLSHGAQRLLCVAVGKLDRPPDLTDDGRNLWLNIGGEE
X_laevis      SLPNELLEKETMLATLGKMLVILNKLLEYQT-MNSIVLSPDSSVCATF--AIKHVLASPVKRLLCINIGDLGILHEFDNDGEYVCLNICGTY

                     560       570       580       590       600       610       620       630       640
D_rerio       TEDKCVNRLTLCLREGESLTAGFMQALLGLILPVAYEFNPALVLGIVEETAAKTRLMRVWGHMTCLIQGLARGRMLTLLQGYDKDLLEL-TV
H_sapiens     AAALS---MFHVSTPLPVMTGGFLSCILGLVLPLAYGFQPDLVLVALGPGHGL--QGPHAALLAAMLRGLAGGRVLALLEENSTPQLAGILA
M_musculus    AAIQS---MFHFSTPLPQTTGGFLSLILGLVLPLAYGFQPDMVLMALGPAHGL--QNAQAALLAAMLRSPVGGRILAVVEEESIRLLARSLA
P_troglodytes AAALS---MFHVSTPLPVMTGGFLSCVLGLVLPLAYGFQPDLVLVALGPGHGL--QGPHAALLAAMLRGLAGGRVLALLEEDSTPQLAGILA
S_scrofa      -GRWG---GLSGRRGVPWTTGGFLSCVLALVLPLAYSFQPDLVLVVLGPAHGL--QDPQAALLAALLRGPAGGRVLALVDEESTPQLAVVLA
B_taurus      AAAPS---MFHVSVPLPVTTGGFLSCALALVLPLAYSFQPDLVLVALGPAHGL--RDPQAALLAALLRGPAGGRVFALVDEESTPQLATVLA
O_aries       ATAPS---IFHVSVPLPVTPGGFLSCVLALVLPLAYSFQPDLVLVALGPGHGL--RDPQAALLAALLRGPAGGRVFALVDEESTPQLATVLA
X_laevis      PTGMSNRQIYLKWTESPNEYSSFFYIIFCCILPLAYNYQPDFIIITTGSNRTI--GDKDISLLISLLQGLANGRILTIIPETEPKLGQKLVK

                   650       660       670       680       690       700
D_rerio       SALSGASISPLGPLRAPKPEDVEMMEKQRQRLQERWGLLRCTVSESW-------------
H_sapiens     RVLNGEAPPSLGPSSVASPEDVQALMYLRGQLEPQWKMLQCHPHLVA-------------
M_musculus    QALHGETPPSLGPFSKATPEEIQALMFLKARLEARWKLLQVAAPPP--------------
P_troglodytes RVLNGEAPPSLGPSSVASPEDVQALMYLRGQLEPQWKMLQCHPHLVA-------------
S_scrofa      RVLHGEAPPSLGPFCMASLETMQALMHLRGQLEPQWKMLRVAGETGR--PGSA-------
B_taurus      RVLNGEAPPSLGPFSMAAPEDTQALMYLRGRLEPRWKMLQVAAPH---------------
O_aries       RVLNGEAPPSLGPFSMAAPEDTQALMYLRGRLEPRWKMLQVAGPPG--------------
X_laevis      CLSDSPGEKHFGPHRAPSLENIQNLKEKLDIIEKEWKMLQCSAKAAGTIPGSTHQCETRD
```

Supplementary Fig. 1. Sequence alignment of HDAC10 orthologues. Sequence alignment prepared with Clustal Omega, figure prepared with Jalview; the numbering scheme is that of *D. rerio* HDAC10 (zHDAC10). The glutamate gatekeeper conserved in all HDAC10 orthologues is highlighted in red (E274 in zHDAC10). The universal 3_{10} helix ηA2 insertion in loop L1 that further constricts the HDAC10 active site is highlighted in purple (P^{23}ACE in zHDAC10). Conserved negatively charged surface residues surrounding the active site are highlighted in orange, and all other conserved residues are highlighted in blue. Note that the N-terminal PDAC catalytic domain is highly conserved in vertebrates, whereas the C-terminal ΨDAC domain is much more divergent.

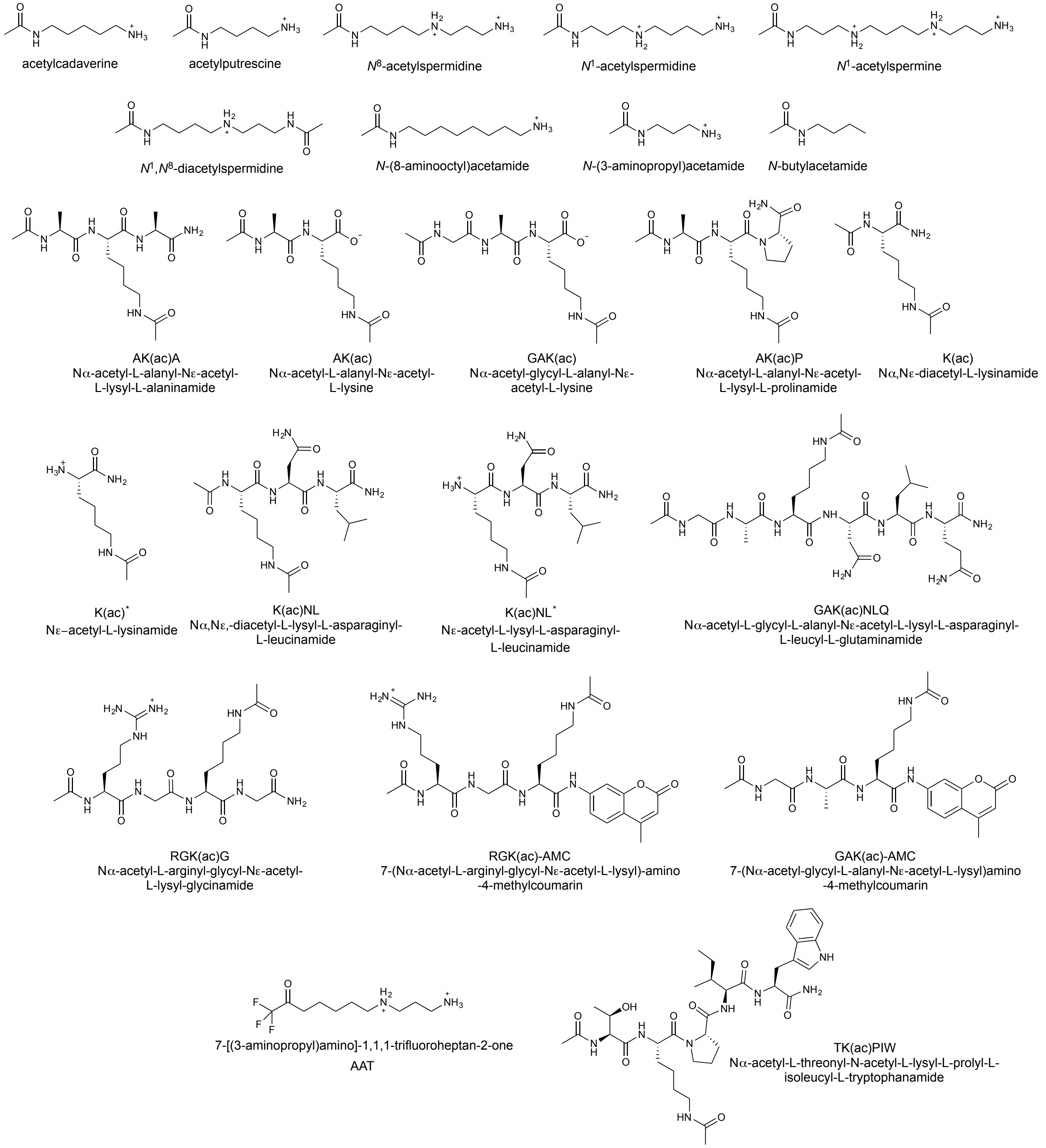

Supplementary Fig. 2. Substrate and inhibitor structures.

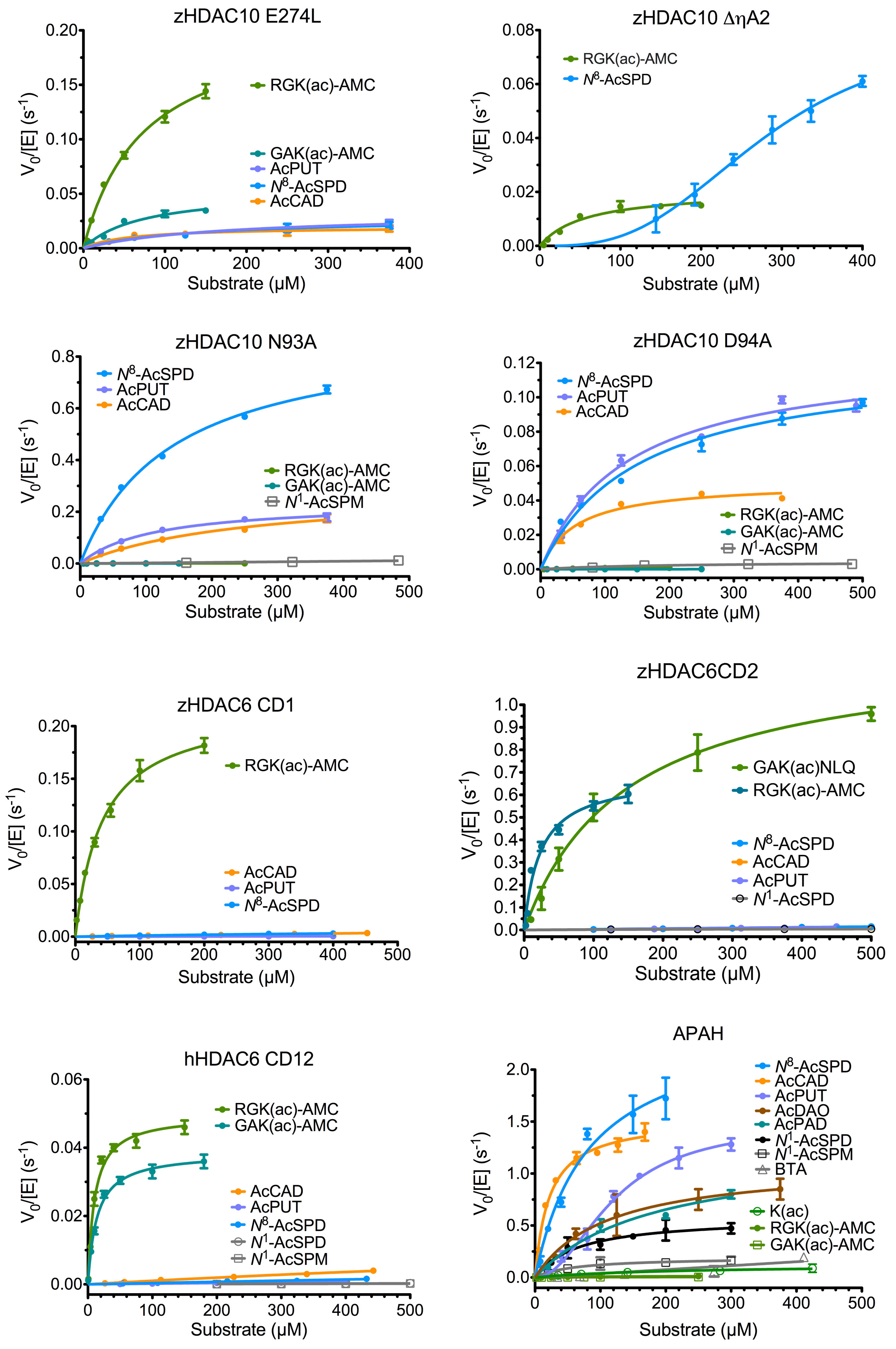
zHDAC10 E274L
RGK(ac)-AMC
GAK(ac)-AMC
AcPUT
N8-AcSPD
AcCAD
V0/[E] (s-1)
Substrate (μM)
zHDAC10 ΔηA2
RGK(ac)-AMC
N8-AcSPD
V0/[E] (s-1)
Substrate (μM)
zHDAC10 N93A
N8-AcSPD
AcPUT
AcCAD
RGK(ac)-AMC
GAK(ac)-AMC
N1-AcSPM
V0/[E] (s-1)
Substrate (μM)
zHDAC10 D94A
N8-AcSPD
AcPUT
AcCAD
RGK(ac)-AMC
GAK(ac)-AMC
N1-AcSPM
V0/[E] (s-1)
Substrate (μM)
zHDAC6 CD1
RGK(ac)-AMC
AcCAD
AcPUT
N8-AcSPD
V0/[E] (s-1)
Substrate (μM)
zHDAC6CD2
GAK(ac)NLQ
RGK(ac)-AMC
N8-AcSPD
AcCAD
AcPUT
N1-AcSPD
V0/[E] (s-1)
Substrate (μM)
hHDAC6 CD12
RGK(ac)-AMC
GAK(ac)-AMC
AcCAD
AcPUT
N8-AcSPD
N1-AcSPD
N1-AcSPM
V0/[E] (s-1)
Substrate (μM)
APAH
N8-AcSPD
AcCAD
AcPUT
AcDAO
AcPAD
N1-AcSPD
N1-AcSPM
BTA
K(ac)
RGK(ac)-AMC
GAK(ac)-AMC
V0/[E] (s-1)
Substrate (μM)

Supplementary Fig. 3. Steady-state kinetics of APAH, HDAC10 mutants, and HDAC6. Data represent mean ± s.d. (n = 3). Abbreviations: AcCAD, acetylcadaverine; AcPUT, actylputrescine; AcSPD, acetylspermidine; AcSPM, acetylspermine.

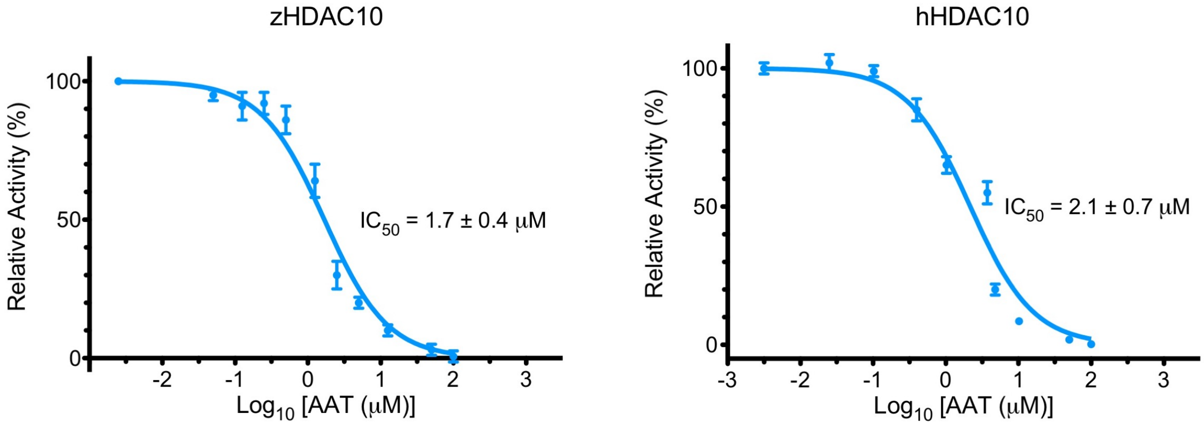

Supplementary Fig. 4. Inhibitory potencies of AAT against zHDAC10 and hHDAC10. Experimental approach described in Methods; the concentrations of zHDAC10, hHDAC10, and N^8-acetylspermidine were 0.49 μM, 0.50 μM, and 192.2 μM, respectively. Using the Cheng-Prusoff equation, K_i values calculated for AAT against zHDAC10 and hHDAC10 are each 0.7 ± 0.2 μM. Data represent mean ± s.d. (n = 3).

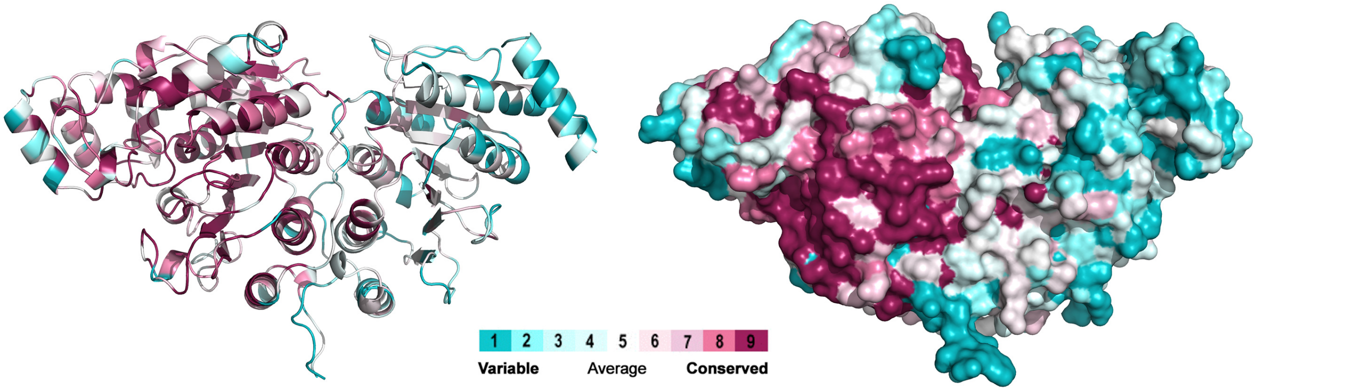

(a)

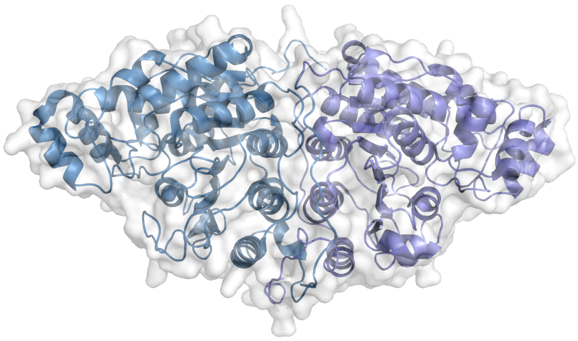

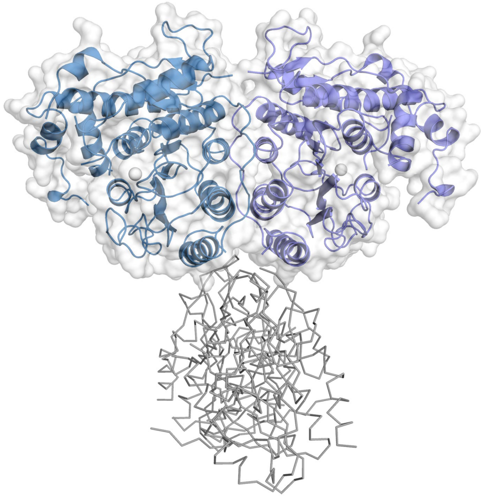

(b) **(c)**

Supplementary Fig. 5. Residue conservation and domain conservation. (a) Residue conservation of 250 HDAC10 orthologues (NCBI Protein Reference Sequences) mapped onto the crystal structure of zHDAC10 using Consurf (http://consurf.tau.ac.il). The butterfly-like architecture of HDAC10 is stabilized by a conserved domain-domain interface; the PDAC domain exhibits much more amino acid sequence conservation than the ΨDAC domain, which is much more variable. **(b)** The highly conserved domain-domain interface is highly similar to HDAC6 (blue for CD1 and purple for CD2; PDB 5G0J). **(c)** This interface may originate from an ancestral HDAC homodimer, as exemplified by the homodimeric HDAC domains of Clr3 (blue/purple) from *Schizosaccharomyces pombe* (PDB 5IKK). The Arb2 (Argonaute binding protein 2) domain of Clr3 is shown as a gray Cα trace.

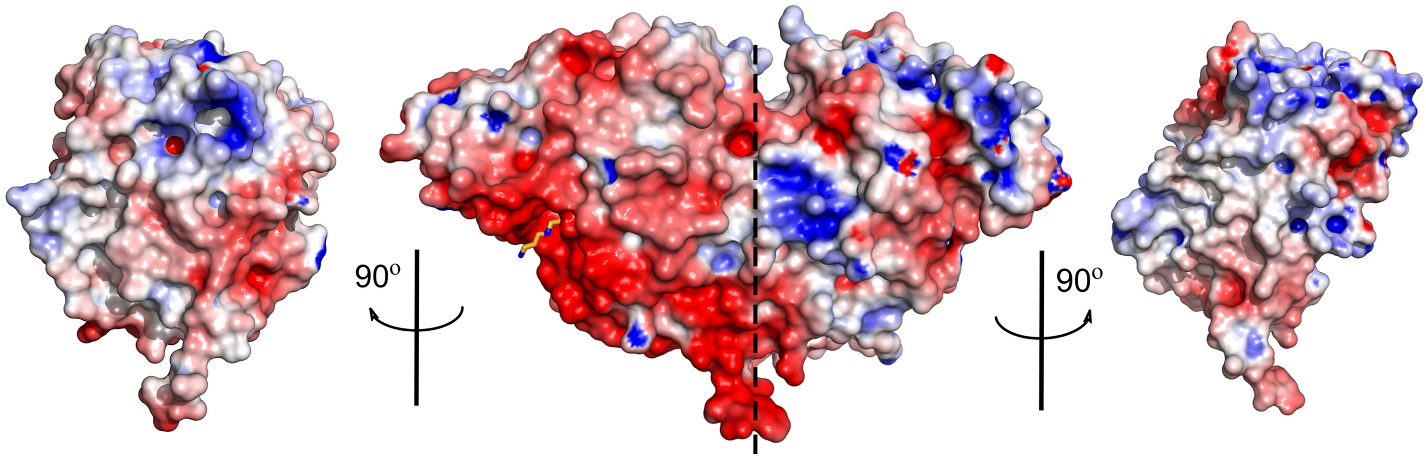

Supplementary Fig. 6. Electrostatic surface potential. Electrostatic potential (-5 kT–5kT, red–blue) mapped onto the molecular surface of zHDAC10. In the center view, the PDAC domain is on the left-hand side and the ΨDAC domain is on the right-hand side. The active site in the PDAC domain is highly anionic and the PDAC-ΨDAC domain interface is highly hydrophobic. The transition state analogue inhibitor AAT is shown as a stick-figure (C = yellow, N = blue) to indicate the location of the active site. The active site tunnel is surrounded by negative charge, which is complementary to the positive charge of the acetylpolyamine substrate.

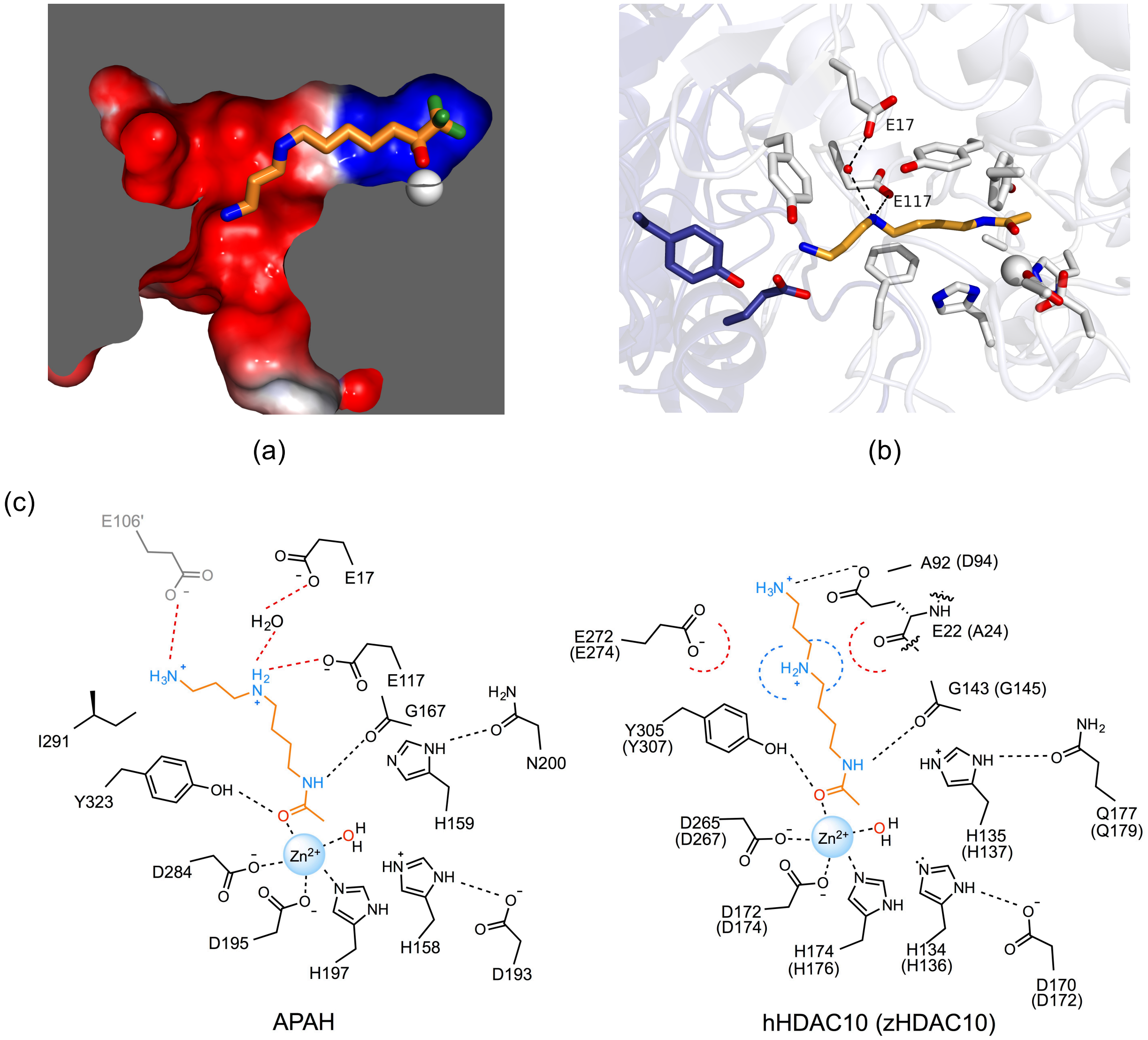

Supplementary Fig. 7. Active site of bacterial APAH and comparison with HDAC10. (a) The active site of APAH is characterized by an "L-shaped" tunnel with electrostatic potential mapped onto the molecular surface (-5 kT–5 kT, red–blue). The transition state analogue inhibitor AAT is shown as a stick figure and the catalytic Zn^{2+} ion is shown as a white sphere (PDB 4ZUM). **(b)** Intermolecular interactions observed in the crystal structure of H159A APAH complexed with the intact substrate N^8-acetylspermidine (PDB 3Q9C). The N4 amino group of N^8-acetylspermidine is recognized by a direct hydrogen bond with E117 (3.2 Å). **(c)** Intermolecular interactions of N^8-acetylspermidine in the active site of hHDAC10 based on the structure of the zHDAC10-AAT complex. Charge-charge interactions between enzyme and substrate that are not sufficiently close to be characterized as hydrogen bonds are indicated by dotted semicircles.
